# Critical role for a promoter discriminator in RpoS control of virulence in *Edwardsiella piscicida*

**DOI:** 10.1371/journal.ppat.1007272

**Published:** 2018-08-31

**Authors:** Kaiyu Yin, Yunpeng Guan, Ruiqing Ma, Lifan Wei, Bing Liu, Xiaohong Liu, Xiangshan Zhou, Yue Ma, Yuanxing Zhang, Matthew K. Waldor, Qiyao Wang

**Affiliations:** 1 State Key Laboratory of Bioreactor Engineering, East China University of Science and Technology, Shanghai, China; 2 Laboratory for Marine Fisheries Science and Food Production Processes, Qingdao National Laboratory for Marine Science and Technology, Qingdao, China; 3 Shanghai Engineering Research Center of Maricultured Animal Vaccines, East China University of Science and Technology, Shanghai, China; 4 Institut de Biotecnologia i Biomedicina, Dept. de Bioquímica i Biologia Molecular, Universitat Autònoma de Barcelona, Barcelona, Spain; 5 Division of Infectious Diseases, Brigham and Women's Hospital and Harvard Medical School, Boston, Massachusetts, United States of America; McMaster University, CANADA

## Abstract

*Edwardsiella piscicida* is a leading fish pathogen that causes significant economic loses in the aquaculture industry. The pathogen depends on type III and type VI secretion systems (T3/T6SS) for growth and virulence in fish and the expression of both systems is controlled by the EsrB transcription activator. Here, we performed a Tn-seq-based screen to uncover factors that govern *esrB* expression. Unexpectedly, we discovered that RpoS antagonizes *esrB* expression and thereby inhibits production of *E*. *piscicida’s* T3/T6SS. Using *in vitro* transcription assays, we showed that RpoS can block RpoD-mediated transcription of *esrB*. ChIP-seq- and RNA-seq-based profiling, as well as mutational and biochemical analyses revealed that RpoS-repressed promoters contain a -6G in their respective discriminator sequences; moreover, this -6G proved critical for RpoS to inhibit *esrB* expression. Mutation of the RpoS R99 residue, an amino acid that molecular modeling predicts interacts with -6G in the *esrB* discriminator, abolished RpoS’ capacity for repression. In a turbot model, an *rpoS* deletion mutant was attenuated early but not late in infection, whereas a mutant expressing RpoS^R99A^ exhibited elevated fitness throughout the infection period. Collectively, these findings deepen our understanding of how RpoS can inhibit gene expression and demonstrate the temporal variation in the requirement for this sigma factor during infection.

## Author summary

*Edwardsiella piscicida*, a major fish pathogen, relies on T3/T6SSs for virulence and the EsrB transcription activator promotes the expression of these secretion systems and many other genes that enable growth in fish. Here, we found that the alternative sigma factor RpoS inhibits expression of *esrB* thereby diminishing expression of virulence-associated genes. Transcriptome profiling revealed that, as in many other organisms, RpoS enables expression of hundreds of genes, many of which are linked to stress responses, suggesting that RpoS may mediate a trade-off between stress adaptation and virulence. Consistent with this idea, we found that an *rpoS* mutant was attenuated early, but not late in infection of turbot, whereas an *esrB* mutant was attenuated late and not early in infection. Molecular analyses demonstrated that RpoS inhibition of *esrB* expression involves a direct interaction between RpoS and the *esrB* promoter; in particular, interactions between RpoS residue R99 and the -6G nucleotide in the *esrB* promoter discriminator appear to be critical for repression of *esrB* expression. These findings provide new insight into how a sigma factor can impede transcription and demonstrate the temporal dynamics of the requirement for a sigma factor during the course of infection.

## Introduction

*Edwardsiella piscicida* (formerly included in *Edwardsiella tarda*) belongs to the enterobacteriaceae family [1] and is phylogenetically related to *Salmonella enterica* [2]. Like some species of *Salmonella*, *E*. *piscicida* can also infect a broad range of animal hosts including, fish, amphibians, mammals and humans [3]. The organism is a bane of the aquaculture industry because it infects over 20 species of fish, including important farmed species such as turbot, flounder, eel and catfish, resulting in significant economic losses globally [4–6]. Several *E*. *piscicida* virulence determinants, such as adhesins, siderophores, and hemolysin EthA have been uncovered using single mutants (reviewed in [7]) and in genome-wide transposon insertion sequencing (Tn-seq)-based studies [8].

Like *S*. *enterica*, *E*. *piscicida* can grow intracellularly [9–10]. The pathogen relies on its type III and type VI secretion systems (T3/T6SSs) to translocate a repertoire of ~20 putative and known effectors into host cells to occupy this niche [11–13]. Genome-wide analysis revealed that, among the 33 putative two-component system (TCS) encoded in the *E*. *piscicida* genome, EsrA-EsrB is indispensable for *E*. *piscicida* pathogenicity. This TCS controls the expression of the pathogen’s T3/T6SS machineries and their respective effectors, as well as the expression of an additional ~990 genes, some of which have roles during infection [13–14]. For example, EsrB-activated genes are associated with iron sequestration and uptake (hemin uptake and siderophore-mediated iron uptake systems), while genes for basal metabolism were directly downregulated by EsrB [13].

Although the host signals that activate the EsrA-EsrB TCS are unknown, several regulators, including EsrB, PhoP [14], PhoR, and Fur [15], are known to modulate expression of *esrB*. Mutation of *esrAB* has been a fruitful strategy for development of live attenuated vaccines against edwardsiellosis in fish [16–17]. Furthermore, in *S*. *enterica*, the EsrAB homologs SsrAB play a critical role in regulating virulence, and homologs in *Sodalis glossinidius* facilitate its endosymbiont lifestyle [18]. Thus, a systematic dissection of the upstream and downstream regulatory networks in which EsrAB is embedded will further our understanding of *E*. *piscicida* pathogenicity. This knowledge will also potentially facilitate vaccine development as well expand our knowledge of the evolution of signal transduction networks that govern virulence in diverse Gram-negative pathogens.

Here, we used a Tn-seq-based screen to identify upstream regulators of *esrB* expression. Surprisingly, we found that the alternative sigma factor RpoS (σ^S/38^) inhibits *esrB* expression and thus exerts negative control over the expression of *E*. *piscicida’s* T3/T6SSs. RpoS, like other sigma factors, associates with core RNA polymerase (RNAP or E), enabling RNAP promoter recognition during initiation of transcription [19–21]. In many Gram-negative bacteria, RpoS enables transcription of genes associated with the general stress response and stationary phase metabolism [22–25] and our work suggests that is the case in *E*. *piscicida*, as well. While RpoS is also known to negatively regulate gene expression [26–28], there have been few studies of the mechanisms by which RpoS can inhibit transcription [29]. Using a variety of approaches to investigate how RpoS represses *esrB* transcription we found that it can antagonize RpoD (sigma factor 70)-mediated transcription of *esrB*. Notably, the presence of the -6G in the *esrB* promoter discriminator (a sequence found between the -10 element and the transcription start site [30]) was required for RpoS’ repressor function. RpoS R99, a residue predicted to interact with -6G, was required for this sigma factor to inhibit transcription of *esrB*, but RpoS^R99A^ still enabled transcription from other promoters. Finally, studies in a turbot infection model indicate that the requirement for RpoS varies during the course of infection.

## Results

### A Tn-seq-based screen identifies novel regulators of the response regulator EsrB

**Fig 1:**
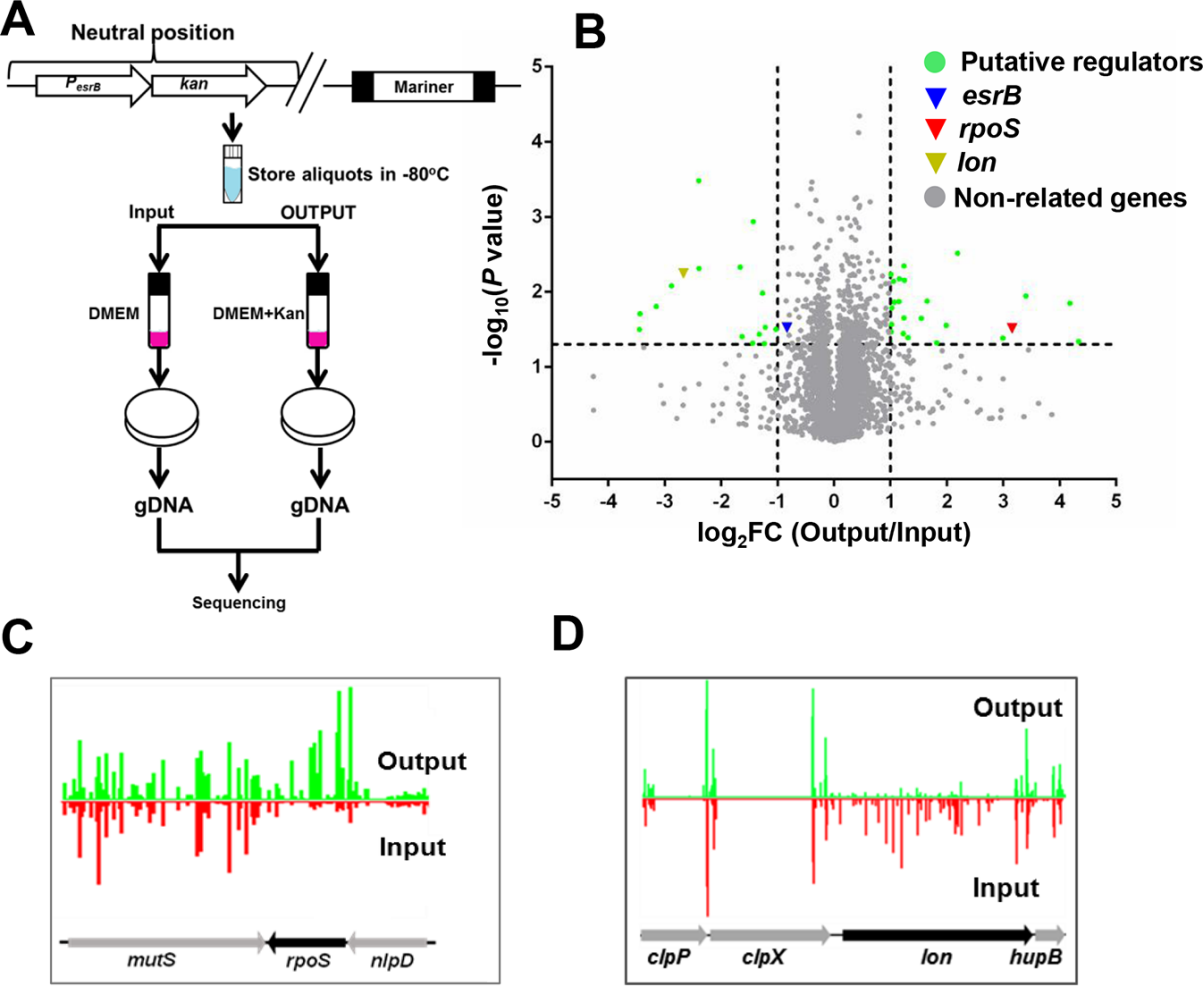
Identification of factors regulating the expression of the response regulator EsrB with a transposon insertion sequencing (Tn-seq) screen. **(A)** Schematic of Tn-seq strategy used for finding putative *esrB* regulators. A transposon library was created in a strain harboring a P_*esrB*_-Kan fusion (inserted in a neutral position on the chromosome), which yields Kan resistance when the *esrB* promoter is active. The library was grown in DMEM conditions, which induces the *esrB* promoter, in either the absence or presence of Kan. The sites and abundance of transposon insertions under the two conditions were compared. **(B)** Volcano plot showing the output/input fold change (FC) in reads of genes as revealed by Tn-seq analysis. The genes of interest were highlighted with cut-off of log_2_ FC > 1 or FC < -1, and *P* value < 0.05 (a measure of the concordance of hits throughout the TA sites in a gene). **(C-D)** Artemis plots of the abundance of reads in *rpoS* (D) and *lon* (E) in DMEM (red) versus DMEM-Kan (green) medium. The height of the red and green bars correlates with the number of reads.

Since EsrB is a key regulator of T3SS and T6SS in *E*. *piscicida*, we devised a Tn-seq [8] based screen to identify genes that control its expression. Initially, we created a reporter of the *esrB* promoter, by fusing the 500 bp segment located upstream of the *esrB* start codon to a kanamycin (Kan) resistance gene (yielding P_*esrB*_-*kan*). This reporter was introduced into a neutral site (between *glms* and ETAE_3537) on the *E*. *piscicida* strain EIB202 chromosome (Fig 1A). Previous studies showed that introduction of DNA into this site does not alter growth [8]. Then, we created a high-density Himar [31] transposon insertion library in this strain (WT::P_*esrB*_-*kan*). The library was cultured in DMEM, a medium that induces the expression of EsrB [13], in either the absence (input) or presence (output) of Kan (Fig 1A). High-throughput sequencing was used to identify the sites and enumerate the frequency of insertions in the input and output libraries. Mutants that are present in the input but not the output library should in principle contain insertions in loci critical for EsrB expression; conversely, mutants that are present at greater frequency in the output, represent insertions in genes that ordinarily repress EsrB expression.

To estimate an optimal concentration of Kan to use for screening the library, we compared the minimum inhibitory concentrations of WT::P_*esrB*_-*kan* and a derivative with a deletion of *esrB* (Δ*esrB*::P_*esrB*_-*kan*). The latter strain was used because EsrB is known to promote *esrB* expression [14]. The two strains had similar growth in the absence of Kan, but with increasing concentrations of Kan the Δ*esrB*::P_*esrB*_-*kan* strain exhibited progressive growth defects relative to WT::P_*esrB*_-*kan* (S1A Fig). At a Kan concentration of 600 μg/ml, growth of the WT::P_*esrB*_-*kan* strain was not decreased, whereas growth of the Δ*esrB*::P_*esrB*_*-kan* strain was markedly inhibited; thus, Kan 600 μg/ml was used for the screen.

A plot of the percentage of TA sites disrupted per gene vs the frequency of genes showed that the input library had a high degree of saturation, where the majority of non-essential genes had ~60% of TA sites disrupted (S1B Fig) [8]. We compared the transposon distribution profiles in the input and output libraries with the Con-ARTIST pipeline [32] to identify genes that were either under- or over-represented (|log_2_(FC)| > 1 and *P* < 0.05) in the output library; such ‘conditionally depleted’ or ‘conditionally enriched’ genes represent candidate loci that likely promote or inhibit expression of EsrB respectively. There were 23 genes with a greater abundance of insertions in the DMEM+Kan cultures and 16 genes with a diminished abundance of insertions in the Kan-containing medium (Fig 1B, S1 Table and S2 Table). As expected, there were fewer insertions in *esrB* in the output library then in the output, but the difference did not reach the 2-fold threshold. The screen did not yield other known regulators of *esrB* expression such as PhoP and Fur, likely because these genes are required for growth in the conditions used for the screen [8] and thus were not included in our analyses.

To validate a subset of the screen hits, we picked 7 insertion mutants present in a defined EIB202 transposon library created in our lab that were hits in the screen. qRT-PCR was used to measure *esrB* transcript levels in the WT and the mutants and in all cases the results were consistent with the findings from the screen (S1C Fig). These observations suggest that many of the 39 genes identified in the screen play a role modulating the expression of the global regulator, *esrB*. Furthermore, 6 of the hits were in genes encoding hypothetical proteins (S1 Table and S2 Table), suggesting that future studies defining the functions of these proteins will shed light on the pathways controlling *esrB*. However, it is possible that some of the hits, such as in *cpxR* [33], are attributable to the stresses imposed by kanamycin itself. Notably, the abundance of insertions in the gene encoding the RpoS sigma factor (*rpoS*) was greater in the output vs the input library (FC ratio = 8.3), suggesting that RpoS is a repressor of *esrB* (Fig 1C). The diminished abundance of insertions in the gene encoding the ATP-dependent protease Lon in the output library (FC = 0.16) (Fig 1D) is consistent with idea that RpoS inhibits *esrB* expression, since Lon is an established negative regulator of RpoS [34].

### RpoS negatively regulates secretion of T3/T6SS products by inhibiting expression of EsrB

**Fig 2:**
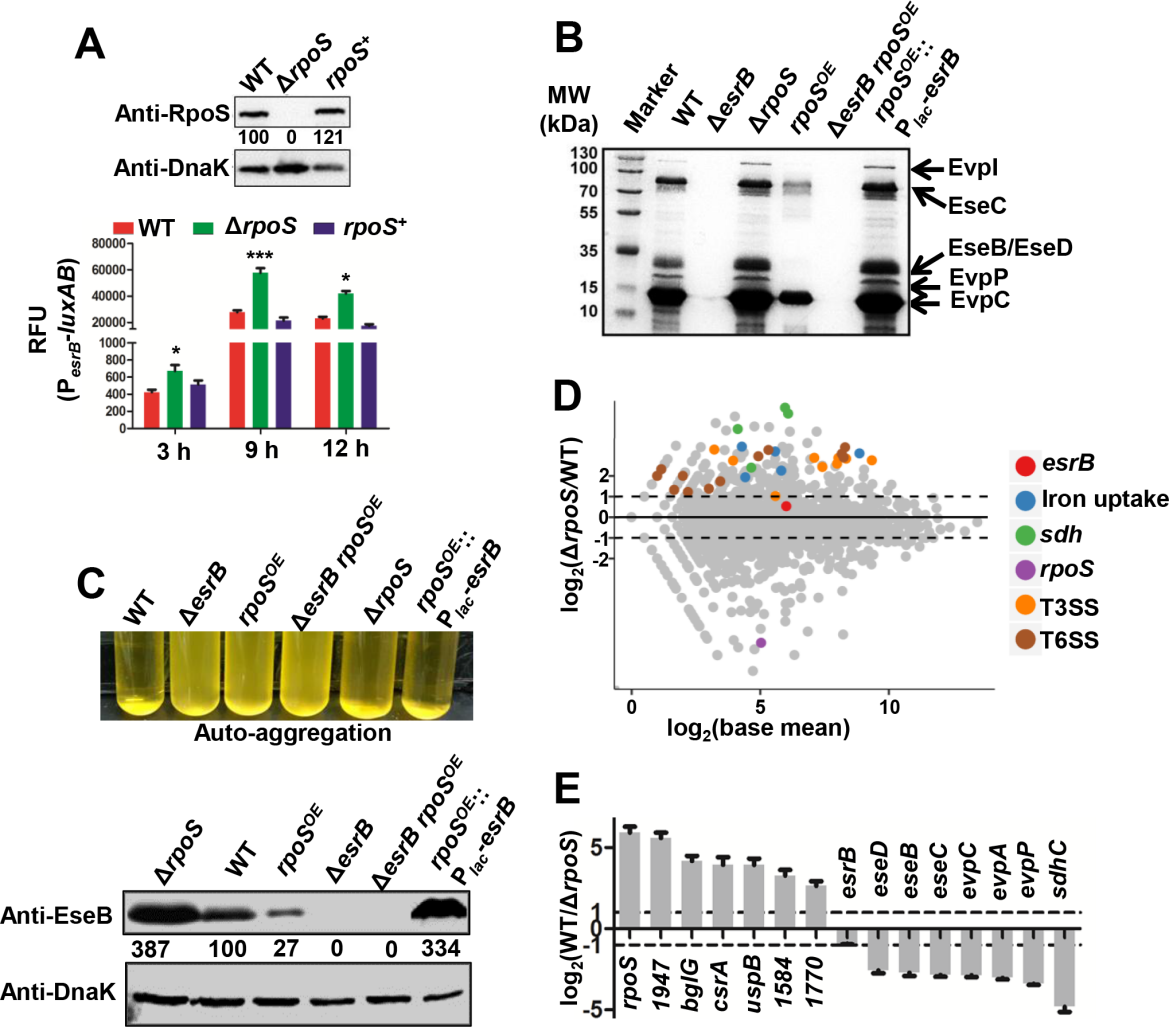
RpoS represses EsrB and T3/T6SS expression. **(A)** Relative fluorescence units (RFU) of the WT, Δ*rpoS* and *rpoS*^*+*^ strains grown in DMEM medium at 3, 9, 12 h after inoculation. The results are shown as the mean ± S.D. (*n* = 3). ***, *P* < 0.0001; *, *P* < 0.01 based on student’s *t*-test. Western blot of RpoS abundance in indicated strains at 9 h is shown on top; DnaK abundance was used as a loading control. The numbers correspond to densitometry measurements. **(B)** Extracellular protein profiles of WT, Δ*esrB*, Δ*rpoS*, *rpoS*^OE^(*rpoS* over-expression), Δ*esrB rpoS*^OE^, and *rpoS*^OE^:: P_*lac*_-*esrB* (*rpoS*^OE^ strain harboring constitutively expressing *esrB* driven from P_*lac*_) were separated on SDS-PAGE gels, and specific bands corresponding to T3SS and T6SS proteins [10, 14] are shown. **(C)** Autoaggregation of the indicated strains statically cultured in DMEM, at 28°C for 24 h. The concentrated supernatants from the same amount of cells were blotted with anti EseB specific antiserum. DnaK was used as the loading control for the blots. **(D)** MA plots showing differences in the transcriptomes of WT and Δ*rpoS* cultured in DMEM (*n* = 3). T3SS, T6SS and iron uptake related genes are highlighted. **(E)** qRT-PCR assays for the indicated transcripts. The results shown are mean ± S.D. (*n* = 3). *gyrB* was used as the internal control.

To further investigate RpoS control of *esrB* expression, we constructed an *rpoS* deletion mutant in the WT::P_*esrB*_-*kan* background (Δ*rpoS*::P_*esrB*_-*kan*), as well as a strain where this deletion was complemented (*rpoS*^+^::P_*esrB*_-*kan*). The Δ*rpoS*::P_*esrB*_-*kan* mutant exhibited significantly higher (minimum inhibition concentration [MIC] of 1200 μg/ml) resistance to Kan than either the WT::P_*esrB*_-*kan* or *rpoS*^+^::P_*esrB*_-*kan* strains (MIC of 600 μg/ml), consistent with the idea that RpoS represses the activity of the *esrB* promoter. Similarly, when the *esrB* promoter was fused to *luxAB* (P_*esrB*_-*luxAB*), enabling *esrB* promoter activity to be measured as fluorescence, there was significantly greater fluorescence detected in the Δ*rpoS* strain than in WT or *rpoS*^+^ complemented strains (Fig 2A), demonstrating that *esrB* promoter activity is inhibited by RpoS, particularly in cells entering stationary phase at 9 h, when RpoS is highly induced [35–36].

The response regulator EsrB is critical for the expression of T3/T6SS in *E*. *piscicida* EIB202 [14]. To begin to assess the consequences of RpoS inhibition of *esrB* expression on T3/T6SS-related functions, we compared the extracellular protein profiles of several strains including WT, Δ*esrB*, Δ*rpoS* and *rpoS*^*OE*^; in the latter strain, *rpoS* is driven by_,_ the promoter for the 30S ribosomal protein, to enhance expression of this sigma factor [2]. As anticipated, T3/T6SS proteins were over-produced in the Δ*rpoS* mutant, while there were reduced yields of T3/T6SS proteins in *rpoS*^*OE*^ compared to the WT strain (Fig 2B). There was no detectable T3/T6SS secreted products when *esrB* was deleted from the *rpoS*^*OE*^ background, providing additional support for the idea that RpoS repression of genes related to T3/T6SS acts through *esrB* (Fig 2B). Moreover, RpoS inhibition of T3/T6SS secretion was circumvented in a strain constitutively expressing *esrB* driven by the P_*lac*_ promoter (*rpoS*^*OE*^::P_*lac*_-*esrB*), indicating that RpoS repression of virulence factor production is dependent on the *esrB* promoter region (Fig 2B). We also tested whether the same set of strains used in Fig 2B exhibited auto-aggregation, a phenotype attributable to production of EseB, a T3SS apparatus protein [37]. The pattern of auto-agglutination and production of EseB in these 6 strains (Fig 2C) mirrored secretion of T3/T6SS products and is consistent with idea that RpoS exerts negative control over *esrB* expression.

We used RNA-seq to elucidate the RpoS regulon in *E*. *piscicida* by comparing the transcriptomes of the WT and Δ*rpoS* strains. Transcripts of 729 genes were differentially (|log_2_(FC)| > 1 and *P* < 0.05) expressed in the two strains, including 532 genes whose transcripts were apparently up-regulated by RpoS and 197 genes whose transcripts were apparently down-regulated by RpoS (2920 genes were not differentially expressed) (Fig 2D, S3 Table and S4 Table). As expected from the results above, many genes in the T3/T6SS gene clusters had higher transcript levels in the absence of *rpoS*, consistent with the idea that their expression is down-regulated by RpoS (Fig 2D, S4 Table); these observations were corroborated with qRT-PCR assays (Fig 2E). As reported in *S*. *enterica* [29], the *sdh* gene cluster was down-regulated by RpoS. Transcripts of genes related to ferric iron uptake were also less abundant in WT vs Δ*rpoS*. Since EsrB is known to activate the ferric iron uptake system [13], this observation is likely also explained by RpoS repression of *esrB* transcription. Collectively, these protein- and mRNA-based assays are all consistent with the idea that RpoS inhibits the expression of T3/T6SS by repressing the expression of EsrB.

### RpoS modulates *esrB* expression in response to environmental stresses

**Fig 3:**
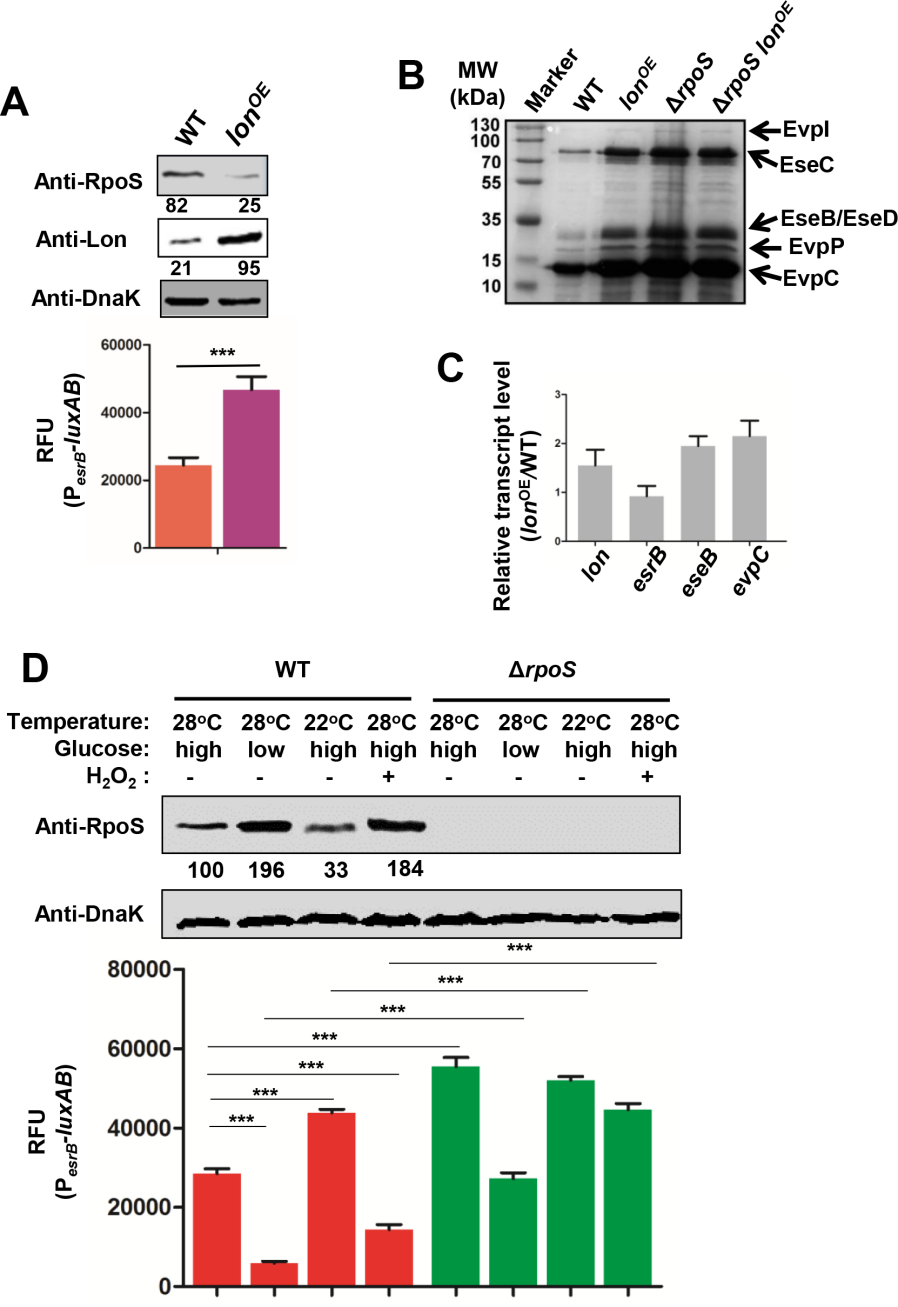
RpoS is regulated by Lon and mediates control of *esrB* expression in response to environmental stresses. **(A)** Western blot analysis of RpoS and Lon (top) and expression of P_*esrB*_*-luxAB* in WT and *lon*^OE^ (Lon over-expression) strains. The results shown are mean ± S.D. (*n* = 3). ***, *P* < 0.0001 based on student’s *t*-test. **(B)** Extracellular protein profiles of WT, *lon*^OE^, Δ*rpoS*, and Δ*rpoS lon*^OE^ were separated on SDS-PAGE gels and specific bands corresponding to T3/T6SS proteins are shown. **(C)** Relative transcript levels of *esrB*, *eseB*, and *evpC* in *lon*^OE^ vs WT strains (qRT-PCR assays with normalization to *gyrB* transcript, mean ± S.D. (*n* = 3). **(D)** Western blot analysis of RpoS and expression of the *esrB* promoter in WT and Δ*rpoS* strains grown in DMEM supplemented with low (1 mg/ml) or high concentrations (4.5 mg/ml) of glucose, with (+) or without (-) addition of H_2_O_2_, and at 22°C or 28°C. DnaK was used as the loading control for the blots. The results shown are mean ± S.D. (*n* = 3). ***, *P* < 0.0001 based on student’s *t*-test.

RpoS production and activity is directly or indirectly regulated through a variety of mechanisms, including the action of the Lon protease [22–25, 34, 38]. Lon over-expression (*lon*^OE^) led to diminished levels of RpoS (Fig 3A, top) and to the concomitant expected elevations in *esrB* transcripts (Fig 3A, bottom) and the amounts of extracellular T3/T6SS proteins and transcripts encoding T3/T6SS proteins EseB and EvpC relative to those detected in the WT (Fig 3B and 3C). Similar amounts of extracellular T3/T6SS proteins were observed in the Δ*rpoS lon*^OE^ as in the Δ*rpoS* strain consistent with the idea that *lon* over-expression modulates T3/T6SS production by depleting RpoS levels.

RpoS is a critical alternative sigma factor involved in the response to a variety of stresses including, starvation, low temperature, and reactive oxygen species (ROS) [22–24]. When we cultured the WT and Δ*rpoS* strains harboring a P_*esrB*_*-luxAB* reporter under various stress conditions, we observed that RpoS levels and P_*esrB*_ activities changed in an inverse fashion (Fig 3D). Taken together, these observations strongly suggest that RpoS mediates a link between environmental conditions and the modulation of expression of *esrB* and its virulence associated regulon.

### RpoS interacts with the *esrB* promoter

**Fig 4:**
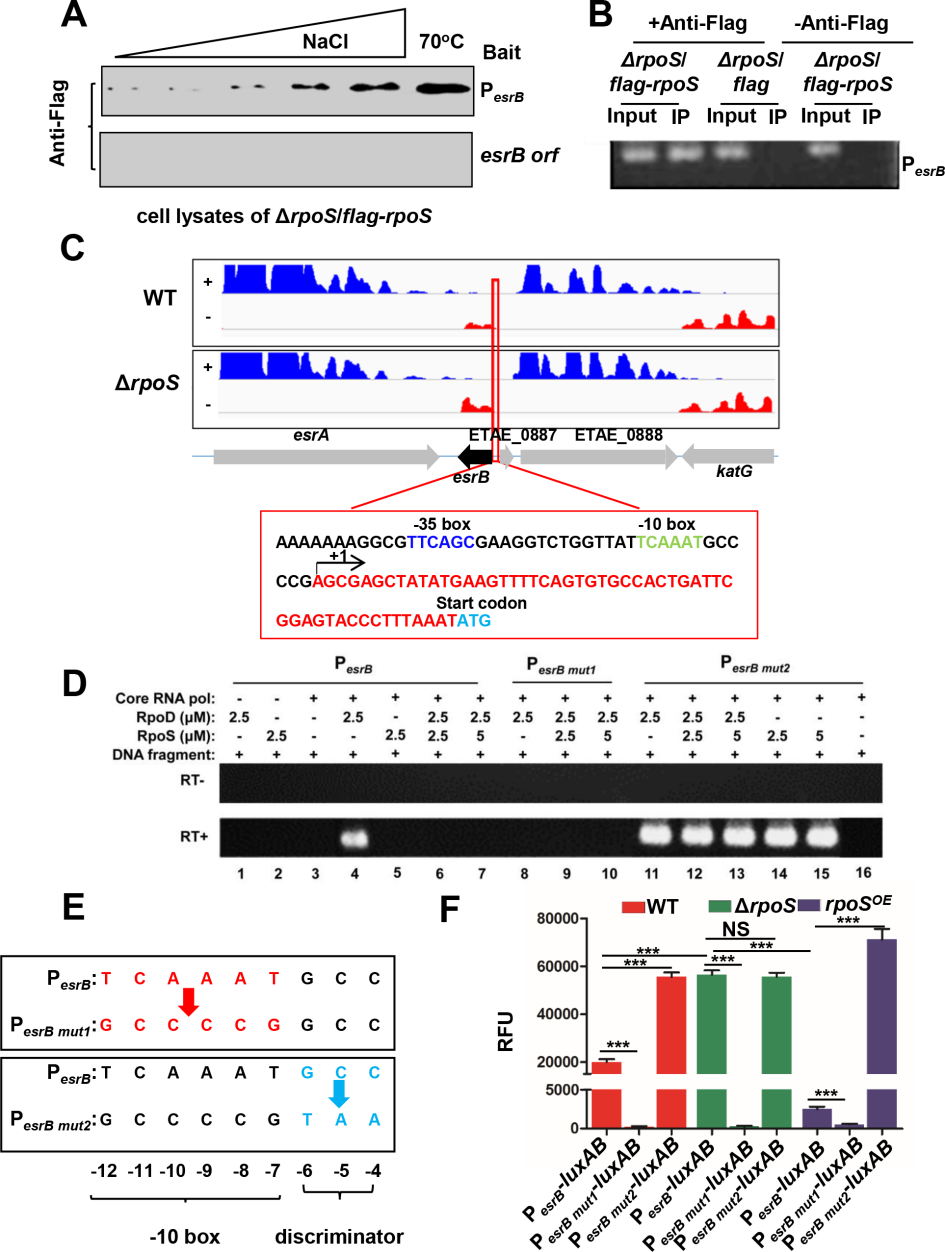
RpoS interacts with the *esrB* promoter region and inhibits *esrB* transcription *in vitro*. **(A)** Pull-down of RpoS by the promoter region of *esrB* (P_*esrB*_). DNA fragments containing P_*esrB*_ or the *esrB* open reading frame (*esrB orf*) were labeled with biotin and fixed to agarose beads. The probe-labeled beads were then mixed with excess Poly (dI:dC) and lysates from Δ*rpoS* cells over-expressing Flag-tagged RpoS (Δ*rpoS/flag-rpoS*), washed, and eluted with a concentration gradient of NaCl and ultimately treated with ddH_2_O at 70°C to release bound proteins, followed by western blot analysis with an anti-Flag-specific monoclonal antibody. **(B)** ChIP assay analysis of RpoS binding to P_*esrB*_ *in vivo*. Stationary phase cells were cross-linked, washed, and sonicated to produce sheared chromosomal DNA. DNA was purified from the sheared pellets both before precipitation (input) and after precipitation in the presence (+) and absence (-) of the anti Flag antibody (IP). PCR was then used to amplify P_*esrB*_. **(C)** Normalized strand-specific-RNA-seq reads of *esrB* transcripts in Δ*rpoS* vs WT were used to identify the +1 site of the *esrB* transcript (depicted with an arrow). **(D)** *In vitro* transcription reactions using templates containing P_*esrB*_, P_*esrB mut1*_ or P_*esrB mut2*_, NTPs, and *E*. *coli* RNAP core enzyme as well as *E*. *piscicida* RpoS and/or RpoD. Transcripts from the reactions were purified, reverse-transcribed (RT, +) and detected using PCR. As a control, the same purified transcripts were treated using the same process but without addition of reverse transcriptase (RT, -). **(E)** Schematic of two engineered variants of the *esrB* promoter; P_*esrB mut1*_ contains substitutions in the -10 box and P_*esrB mut2*_ contains substitutions in the neighboring discriminator GCC sequence. **(F)** Fluorescence values from WT, Δ*rpoS*, and *rpoS*^OE^ strains with chromosomal *luxAB* reporter driven by P_*esrB*_, P_*esrB mut1*_ or P_*esrB mut2*_. *** *P* < 0.0001, NS, non-significance (*P* >0.05) based on student’s *t*-test.

Next we investigated whether RpoS can interact with the *esrB* promoter region. Initially, a pull-down assay, where biotin labeled P_*esrB*_ attached to beads was used as bait, was used to test if RpoS binds this region; beads bound to a biotin labeled portion of the *esrB* open reading frame (*orf*) were used as a negative control. Lysates from a Δ*rpoS* strain expressing a functional (S2A Fig, and S2B Fig) Flag-tagged RpoS (Δ*rpoS/flag-rpoS*) were incubated with the beads and bound proteins were eluted with NaCl. The Flag-tagged RpoS was eluted from the P_*esrB*_ bait sequence but not from the *esrB orf* bait (Fig 4A), showing that RpoS can bind to this promoter.

A chromatin immunoprecipitation assay (ChIP) was performed to investigate whether RpoS binding to P_*esrB*_ could be detected *in vivo*. Protein-cross-linked DNA obtained from Δ*rpoS* cells expressing *flag*-*rpoS* or *flag* alone was immuno-precipitated using an anti Flag-tag antibody. A PCR assay that amplified P_*esrB*_ was carried out on the input and precipitated DNA from both strains. The immunoprecipitate from the strain expressing Flag-tagged RpoS and not from the strain expressing the Flag tag contained the P_*esrB*_ amplification product, whereas the input DNA from both strains contained this product (Fig 4B). In addition, no PCR product was detected in the IP in the absence of the anti-Flag antibody (Fig 4B). Together, these findings strongly suggest that RpoS binds to the *esrB* promoter region in *E*. *piscicida*. We presume that RpoS is binding to the *esrB* promoter as part of RNAP holoenzyme, since sigma factors are not thought to interact with promoters outside of the context of this macromolecular complex [21].

### The *esrB* promoter discriminator sequence is critical for RpoS repression of RpoD driven transcription of *esrB in vitro*

RpoS is a member of the σ^70^ family of proteins, and its binding motif is similar to that of RpoD (σ^70^) [39]. In *E*. *coli* and other bacteria, these two sigma factors bind to overlapping sites in the -10 region of the promoter [29, 40]. The RNA-seq profiles obtained above were used to identify the +1 site of the *esrB* transcript (Fig 4C). As expected, there was greater abundance of *esrB* transcripts in the Δ*rpoS* mutant and the predicted -35 and -10 sequences are similar to known RpoS promoter binding sites [24, 36, 40].

*In vitro* transcription reactions were carried out to begin to dissect the molecular determinants of RpoS repression of *esrB* transcription. For these assays, we used *E*. *coli* core RNAP and *E*. *piscicida* RpoD, with or without the addition of *E*. *piscicida* RpoS in the reaction mixtures. The *esrB* promoter (P_*esrB*_) driving *esrB* was used as the transcription template. The transcripts generated from the different reaction conditions were assessed using reverse transcription (RT) PCR with primers that targeted the *esrB* ORF region. Addition of *E*. *piscicida* RpoD, but not RpoS, to *E*. *coli* core RNAP was sufficient to drive transcription from P_*esrB*_ (Fig 4D, lanes 4–5). Addition of RpoS to the reaction mixtures abolished RpoD-mediated transcription of *esrB* (Fig 4D, lanes 6–7), demonstrating that RpoS is sufficient to repress *esrB* expression driven from P_*esrB*_ but not to mediate transcription from this promoter.

We also engineered mutant forms of the *esrB* promoter to test the importance of two promoter elements in enabling RpoS to inhibit *esrB* transcription. In one mutant, P_*esrB mut1*_, the P_*esrB*_ -10 box AT-rich region, which is thought to be critical for RpoS binding [24, 36, 40], was replaced with CG nucleotides (Fig 4E). An additional variant of P_*esrB*_ was constructed where the discriminator sites (GCC) found immediately downstream of the -10 box were substituted with TAA nucleotides, yielding P_*esrB mut2*_ (Fig 4E). The discriminator region plays a role in proper initiation of transcription and transcription start site selection from σ^70^ dependent promoters [41–42]; furthermore, in *S*. *enterica* serovar Typhimurium the discriminator region of the *sdh* promoter was required for RpoS repression of *sdh* expression [29]. Transcription from the P_*esrB mut1*_ promoter, containing the mutated -10 box, was not detected (Fig 4D, lanes 8–10), an expected result given the likely importance of this sequence for either RpoD or RpoS to bind the promoter. Interestingly, when the template DNA containing the mutation of the discriminator sequence (P_*esrB mut2*_) was used in the reaction, RpoS no longer repressed transcription; in fact, in this setting, RpoS was sufficient to drive transcription in the absence of RpoD (Fig 4D, lanes 11–15). Thus, at least in the *in vitro* context, the sequence of the discriminator region in the *esrB* promoter is critical for determining whether RpoS functions to inhibit or enable *esrB* transcription.

For *in vivo* correlations of these *in vitro* observations, we created *luxAB* reporter genes driven by P_*esrB*_ or its variants and introduced them into a neutral chromosomal position in the WT, Δ*rpoS* and *rpoS*^OE^ strains. Immunoblots established that RpoS abundance was 2–3 fold higher in the *rpoS*^OE^ strain than in the WT strain (S2C Fig). As expected, the fluorescence from the reporter driven by P_*esrB*_ was higher in the absence RpoS and lower when RpoS was overexpressed (Fig 4F). There was little detectable fluorescence in any of the backgrounds from the reporter driven by P_*esrB mut1*_, which is expected since neither RpoD nor RpoS bind to this promoter (Fig 4F) [24, 36, 40]. Fluorescence from the reporter driven by P_*esrB mut2*_ was higher than that driven by P_*esrB*_ in the WT background, a finding which could be attributed to either absence of RpoS repression and/or to RpoS-mediated activation of transcription from this mutant promoter. The former explanation likely accounts for the elevation in the magnitude of expression from this mutant promoter because its fluorescence was unchanged in the Δ*rpoS* background. However, there was elevated P_*esrB mut2*_ activity observed in the strain overexpressing *rpoS*, which may be attributable to RpoS contributing to transcriptional activation in this context (Fig 4F). Taken together, these data are consistent with the idea that the discriminator region (GCC) in the *esrB* promoter, which is not essential for RpoS binding, is important for RpoS to interfere with RpoD-mediated transcription of *esrB*.

### The -6G in the *esrB* promoter discriminator region is critical for RpoS repression

**Fig 5:**
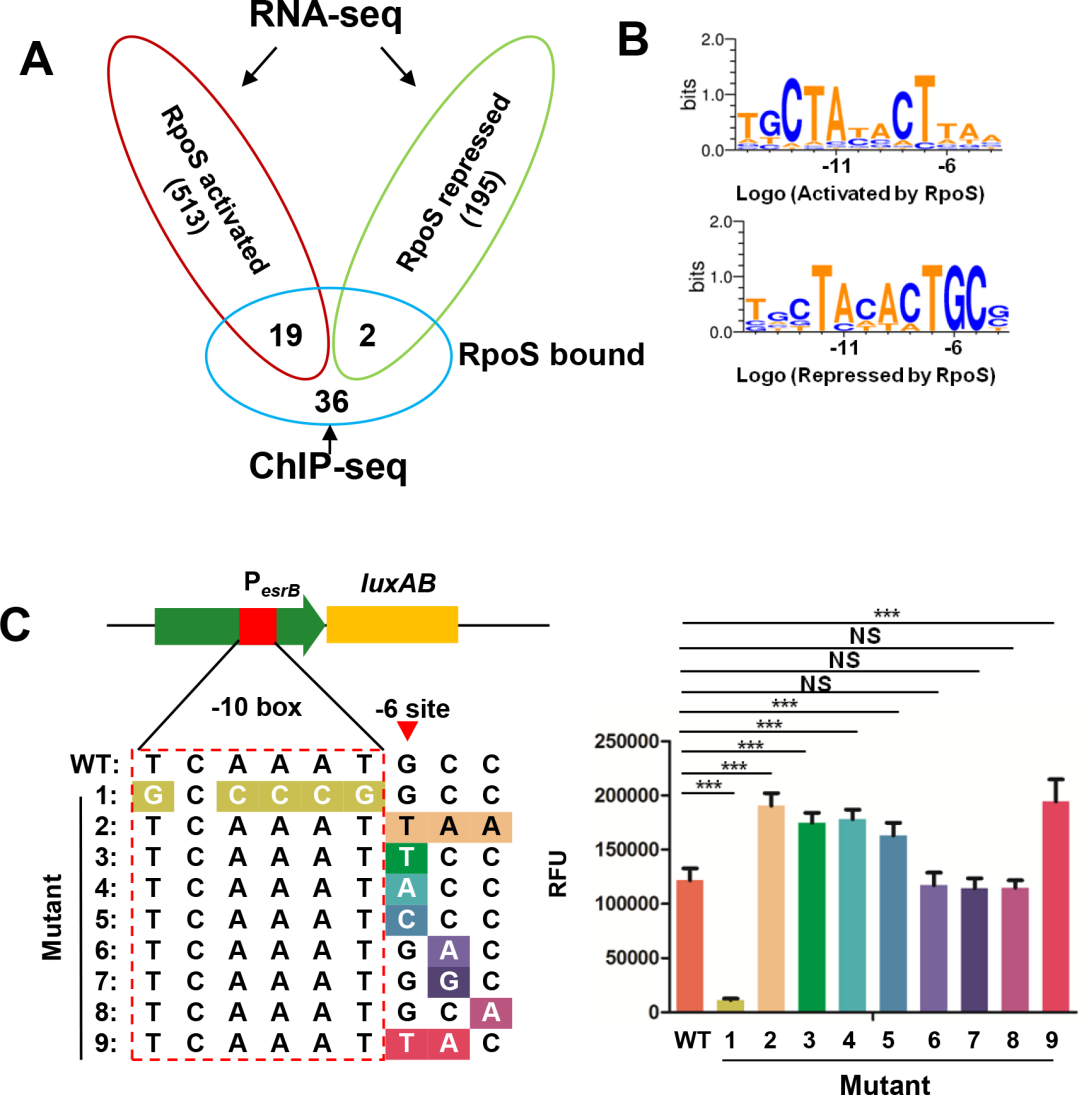
The -6G in the discriminator sequence of RpoS controlled promoters is required for RpoS to act as a repressor. **(A)** Venn diagram showing genes directly bound and regulated by RpoS as revealed by RNA-seq and ChIP-seq analysis. **(B)** RpoS binding motifs on activated and repressed genes. **(C)** A promoterless *luxAB* reporter was fused to WT and mutant P_*esrB*_ (left panel), cloned into plasmid pUTat and introduced into the WT strain. The fluorescence of the respective strains was assayed at 9 h (right panel). *** *P* < 0.0001, NS, non-significance (*P* >0.05) based on student’s *t*-test.

To deepen our understanding of RpoS inhibition of P_*esrB*_ expression, we used ChIP-seq to define the RpoS regulon in stationary-phase *E*. *piscicida* cells grown in DMEM. This analysis revealed that RpoS bound to 57 loci (S5 Table). Besides *esrB*, genes enriched by RpoS ChIP included *rpoS*, *sdhC*, *bglG*, and *mdtJ* (S3A Fig). Using MEME-ChIP [43], a conserved AT-rich RpoS binding motif and a putative -10 box and -35 box were identified (S3B Fig). Combined with the RNA-seq data, these analyses enabled identification of 21 genes whose expression are likely directly regulated by RpoS; *esrB* and *sdhC* were the only candidate targets of direct RpoS repression and 19 candidate RpoS-dependent genes were identified (Fig 5A). We compared the motifs representing the genes activated and repressed by RpoS and found that the -10 box, TAYacT (-12 to -7 sites) were similar, whereas the -6 to -4 sites (relative to -10 box) were distinct in the motifs derived from the activated and repressed genes (Fig 5B). Examination of the RNA-seq data for genes containing the RpoS binding motif (S3B Fig) in their promoter regions revealed an additional 16 candidate genes directly regulated by RpoS (*P* < 0.001). 4 of these genes are putatively repressed and 12 activated by RpoS (S4A Fig and S4B Fig). The RpoS repressed genes usually contain a GCG discriminator sequence whereas the activated genes harbor a distinct and somewhat more variable discriminator sequence (often TAA) (Fig 5B). Notably, all the repressed genes contain a -6G and a -5C (Fig 5B and S4B Fig). Chi-square tests revealed a significant difference (*P* < 0.001) in the occurrence of G and C nucleotides at the -6 and -5 sites of the repressed genes vs the activated genes (not GC) (S4C Fig and. S4D Fig), and in the elevated frequency of G vs non -G in the -6 site in the discriminator region of repressed vs activated genes (*P* < 0.001) (S4E Fig).

The above analyses suggested that the sequence of the discriminator region of RpoS-regulated promoters, in particular the presence of -6G and -5C could determine if RpoS acts as repressor at the respective promoter. To test this idea, we used site-directed mutagenesis to introduce changes in the -6 to -4 sites as well as in the -10 box in the *esrB* promoter region fused to a promoterless *luxAB* reporter. These reporters were introduced into a Δ*esrB* strain and the resulting bioluminescence was measured (Fig 5C). Consistent with the findings in Fig 4F, modifications in the -10 box (Mutant 1) abolished transcription of P_*esrB*_. Substitution of -6G to -6T, A or C alone or together with additional substitutions in -4 and -5 sites all significantly enhanced transcription from P_*esrB*_ (Fig 5C). However, substitutions in the -5 or -4 sites did not alter transcription of P_*esrB*_ as long as there was a -6G (Fig 5C). Similarly, substitution of -6G to T in the promoters of two additional RpoS repressed genes, *sdhC* and 1580 that did not affect the RpoS level in the cells, abolished RpoS repression (S4F Fig and S4G Fig). Taken together, these results demonstrate that the -6G in the discriminator region of RpoS associated promoters is critical for this sigma factor to function as a repressor.

### Arg^99^ is required for RpoS repressor function

**Fig 6:**
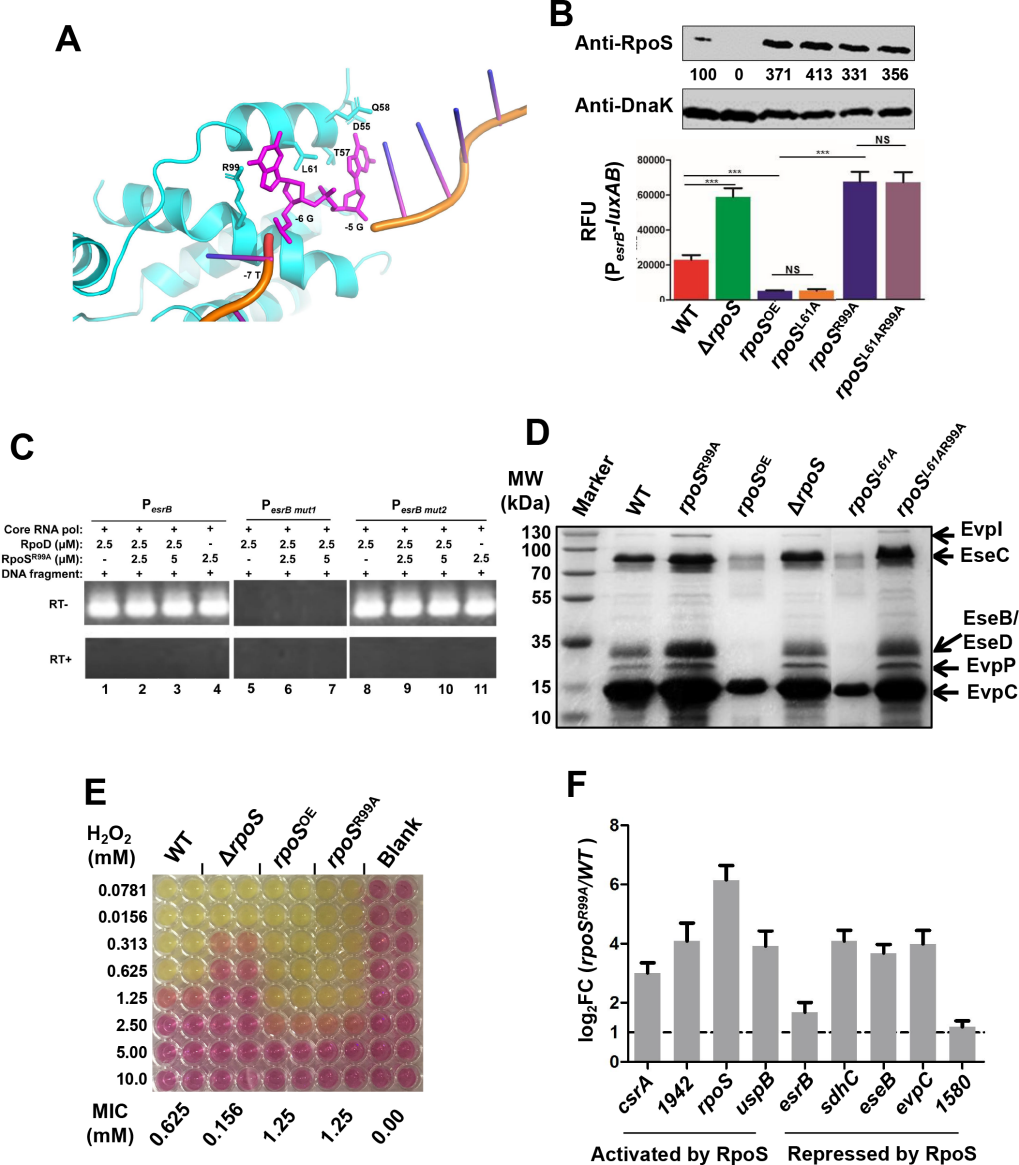
Identification of RpoS R99 as a critical residue for the repression of the expression of *esrB* and other genes. **(A)** Structural model of RpoS interacting with the discriminator sequence based on alignment with of σ^A^ (5VI5, Chain F [44]). **(B)** Fluorescence expressed by P_*esrB*_-*luxAB* in the indicated strain backgrounds. The intracellular RpoS levels of the indicated strains were detected with western blot assays, and DnaK was used as a loading control. The results shown are the mean ± S.D. (*n* = 3). ***, *P* < 0.0001; NS, non-significance (*P* >0.05) based on student’s *t*-test. **(C)** *In vitro* transcription reactions using a P_*esrB*_, P_*esrB mut1*_ or P_*esrB mut2*_ templates, NTPs, and *E*. *coli* RNAP core enzyme as well as *E*. *piscicida* RpoS^R99A^ and RpoD. Transcripts from the reactions were purified, reverse-transcribed (RT, +) and detected using PCR. As a control, the same purified transcripts were treated using the same process but without addition of reverse transcriptase (RT, -). **(D)** RpoS^R99A^ does not repress production of T3/T6SS proteins. **(E)** Growth of indicated strains in increasing concentration of H_2_O_2_. WT, Δ*rpoS*, *rpoS*^OE^, and *rpoS*^R99A^ were inoculated into DMEM containing various concentrations of H_2_O_2_ and statically grown for 24 h at which point OD600 was measured; MICs are shown. **(F)** Capacity of the RpoS^R99A^ mutant to repress or activate genes in the RpoS regulon compared to WT; qRT-PCR analyses with *gyrB* as the internal control. The results shown are the mean ± S.D. (*n* = 3).

We used molecular simulations to model how *E*. *piscicida* RpoS interacts with *esrB* promoter DNA. RpoS was aligned with the *Mycobacterium smegmatis* RNA polymerase sigma factor σ^A^ (5VI5, Chain F) [44]. The alignment (which is close, root-mean-square deviation of 2.065 Å over 215 Cα atoms), places the RpoS DNA binding helices close to the homologous helices in σ^A^. The *E*. *piscicida* RpoS residues R99 and L61 are predicted to be in close proximity with -6G, whereas residues D55, T57, Q58 and L61 are near -5C (Fig 6A). We focused on R99 and L61, and constructed strains overexpressing three RpoS substitution variants, *rpoS*^R99A^, *rpoS*^L61A^, and *rpoS*^L61AR99A^ in the Δ*rpoS* background (Fig 6B, top). The P_*esrB*_-*luxAB* reporter was used to monitor the effects of these mutations on P_*esrB*_ expression. Notably, the R99A substitution abolished RpoS’ capacity to repress P_*esrB*_ expression, but the L61A substitution did not (Fig 6B); similar to RpoS^R99A^, the L61AR99A double substitution did not repress the *esrB* promoter (Fig 6B). Moreover, *in vitro* transcription assays with P_*esrB*_ or its variants (P_*esrB mut1*_ and P_*esrB mut2*_) as the template in a mixture of the RNAP core enzyme, RpoD and RpoS^R99A^ demonstrated that the R99A substitution mutation in RpoS abolished its capacity to repress *esrB* transcription; however, unlike RpoS, RpoS^R99A^ could enable transcription from P_*esrB*_ (Figs 4D and 6C, lanes 1–4). Thus, since RpoS^R99A^ is capable of supporting transcription, its failure to repress transcription from P_*esrB*_ is not simply explained by ablation of its capacity to interact with DNA. Moreover, these results strongly suggest that RpoS R99 interaction with the -6G in the *esrB* promoter discriminator sequence is a critical determinant of whether this sigma factor impedes transcription.

Consistent with the finding that *rpoS*^R99A^ and *rpoS*^L61AR99A^ did not repress *esrB* expression, extracellular levels of T3/T6SS secreted proteins were elevated in strains expressing these RpoS variants (Fig 6D). We compared the capacity of WT, Δ*rpoS*, *rpoS*^OE^, and *rpoS*^R99A^ to resist H_2_O_2_ as a way to begin to compare the capacity of RpoS^R99A^ to activate genes in the RpoS regulon. Interestingly, the MIC of the *rpoS*^R99A^ mutant was higher than WT and identical to that found in *rpoS*^OE^, suggesting that RpoS^R99A^ can still function as an activator (Fig 6E). Moreover, qRT-PCR analyses confirmed that overexpression of RpoS^R99A^ led to elevated transcript levels of genes that are ordinarily RpoS-activated as well as those that are ordinarily repressed by RpoS (Fig 6F). These analyses demonstrate that RpoS^R99A^ retains the ability to activate transcription of genes that are usually upregulated by RpoS, but has lost the capacity to function as a repressor, lending additional support for the idea that the interaction of R99 with -6G is a critical requirement for RpoS to function as a repressor.

### RpoS represses virulence gene expression during infection

**Fig 7:**
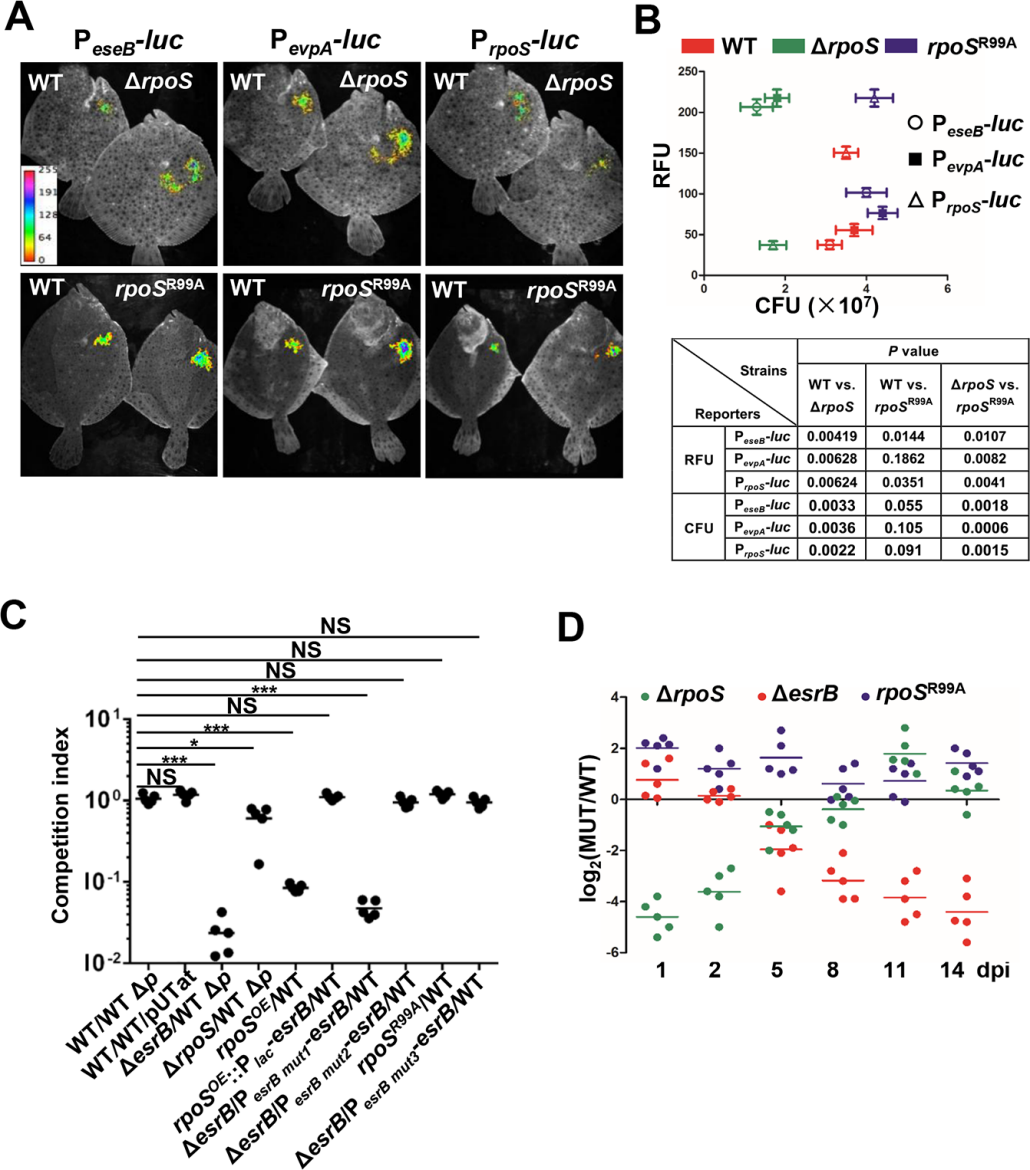
RpoS represses EsrB expression and colonization during infection of the fish. **(A-B)** *In vivo* measurement of T3SS (*eseB*), T6SS (*evpA*) and *rpoS* gene expression using luminescence. The WT, Δ*rpoS*, and *rpoS*^R99A^ strains harboring P_*eseB*_-, P_*evpA*_-, or P_*rpoS*_- *luc* reporter plasmids were inoculated into turbot and luminescence and bacterial burden was measured 8 dpi. The *P* values are based on the ANOVA analysis of the relative fluorescence units (RFU) and the bacterial burden in each fish (*n* = 3). **(C)** *In vivo* competition assays for the Δ*rpoS*, Δ*esrB*, *rpoS*^OE^, and *rpoS*^R99A^ strains vs the WT or WTΔp (WT with pEIB202 cured), which are Cm resistant or sensitive, respectively. The strains *rpoS*^OE^::P_*lac*_-*esrB* (*rpoS*^OE^ constitutively expressing *esrB* driven by P_*lac*_), Δ*esrB/*P_*esrBmut1*_-*esrB*,Δ*esrB/*P_*esrBmut2*_-*esrB*, and Δ*esrB/*P_*esrBmut3*_-*esrB* (Δ*esrB* harboring plasmid pUTat expressing *esrB* driven by P_*esrBmut1*_, P_*esrBmut2*_, or P_*esrBmut3*_) were competed vs the WT. 1:1 mixtures of the indicated strains were i.p. administered into turbot and cultivated for 8 dpi before CFU in the liver were enumerated. *P < 0.01; **P < 0.001; ***, *P*<0.0001, based on ANOVA followed by Bonferroni’s multiple-comparison post-test to compare the data to the values for the corresponding WT/WTΔp samples. **(D)** Competitive indices of the indicated strains mixed with WT or WTΔp inoculated into fish and recovered at 1, 2, 5, 8, 11 and 14 dpi were performed as described in **C** (*n* = 5 per group).

We used *in vivo* fluorescence imaging to investigate RpoS repression of T3/T6SS expression during *E*. *picicida* infection of turbot, a natural host [2]. Luciferase reporters of P_*eseB*_-, P_*evpA*_-, P_*rpoS*_-, and P_*esrB*_- expression were introduced into a neutral position on the chromosome of WT, Δ*rpoS* and *rpoS*^R99A^ strains, and these strains were inoculated i.p. into turbot fish at the same dose and fluorescence was measured 8 days post infection (dpi). The P_*esrB*_-*luc* fusion did not generate sufficient fluorescence for *in vivo* monitoring probably because of the low transcript level of *esrB* (Figs 2E and 4C), but the fusions to the *eseB* and *evpA* promoters, whose expression is activated by EsrB, were sufficiently active and serve as indirect measures of *esrB* expression [13]. As expected, there was little P_*rpoS*_-*luc* activity detected in the Δ*rpoS* background because of RpoS auto-activation; in contrast, and as observed *in vitro* (Fig 7A), there was greater P_*rpoS*_-*luc* activity *in vivo* in the *rpoS*^R99A^ strain than in the WT strain (Fig 7A and 7B). There was significantly greater fluorescence produced by the P_*eseB*_ and P_*evpA*_ fusions in the Δ*rpoS* and the *rpoS*^R99A^ strains than in the WT strain (Fig 7A and 7B). These observations mirror the *in vitro* findings and demonstrate that RpoS represses the EsrB regulon during infection. Furthermore, they show that the RpoS Arg99 residue is required for its repressor activity *in vivo* during infection. Thus, at least at 8 dpi, RpoS negatively regulates *in vivo* virulence factor expression. Despite the elevated virulence gene expression in the Δ*rpoS* mutant, there was ~2x-fewer Δ*rpoS* CFU recovered from infected fish than the WT and the *rpoS*^R99A^ strains (Fig 7B), suggesting that RpoS activated genes may also contribute to *E*. *picicida* growth at some points during infection.

*In vivo* competition experiments were also carried out to elucidate whether RpoS regulation is required for optimal *E*. *piscicida* fitness during infection. The relevant WT comparator strains for these experiments were WT cured of the endogenous R plasmid pEIB202 (WT ΔP), which is known to be proficient at colonization [8], and the WT with an empty stable pUTat (WT/pUTat) [45]. Either of these control strains was inoculated in 1:1 mixtures with different test strains in turbot fish. Each of these strains grew equivalently as assessed in *in vitro* competition assays in LB (S5 Fig). The ratios of the strain mixtures in livers, the organ with the most robust colonization, were determined 8 dpi. As previously observed [8], Δ*esrB* was markedly outcompeted *in vivo*. The Δ*rpoS* mutant had a modest (~2.5 fold) but significant colonization defect (Fig 7C), suggesting either that over-expression of RpoS repressed genes or absence of expression of RpoS-activated genes is detrimental for optimum growth *in vivo*. The *in vivo* growth of *rpoS*^OE^, the strain over-expressing RpoS, was more severely attenuated than the Δ*rpoS* strain (Fig 7C), consistent with the idea that the relief of RpoS repression of the EsrB virulence regulon is critical for the pathogen to grow *in vivo*. Together, these experiments reveal that RpoS regulation is necessary for *E*. *piscicida* optimal growth *in vivo*.

Additional *in vivo* competition experiments were carried out to more directly assess whether RpoS control of EsrB expression contributes to *E*. *piscicida* fitness *in vivo*. A strain constitutively expressing EsrB in the *rpoS*^OE^ background (*rpoS*^OE^::P_*lac*_*-esrB*) competed equally with the WT (CI~1, Fig 7C), strongly suggesting that the enhanced repression of *esrB* in *rpoS*^OE^ accounts for the attenuation of this strain. Conversely, a strain where the native *esrB* promoter was substituted with P_*esrB mut1*_ (Δ*esrB*/P_*esrB mut1*_*-esrB*) exhibited a colonization defect similar to that exhibited by the Δ*esrB* mutant; this observation is consistent with observations shown above (Fig 4D) that this promoter does not support *esrB* expression. However, the strains containing P_*esrB mut2*_ or P_*esrB mut3*_, both of which support *esrB* expression (Figs 4D and 5C), substituted for the native *esrB* promoter (Δ*esrB*/P_*esrB mut2*_*-esrB* and Δ*esrB*/P_*esrB mut3*_*-esrB* respectively) showed no colonization defects (Fig 7C). Notably, the *in vivo* colonization of the *rpoS*^R99A^ strain was comparable to that of the WT at 8 dpi. Coupled with the results shown in Fig 7A, these observations provide strong support for the idea that relief of RpoS-mediated repression of *esrB* expression, and consequent expression of the EsrB regulon (e.g. T3/T6SS expression) is critical for *E*. *piscicida* growth *in vivo*.

### Inverse patterns in the temporal requirements for RpoS and EsrB during chronic infection

*E*. *pisicicida* can cause chronic infections in turbot and during the course of such infections the genetic requirements for fitness are dynamic [8, 13, 16]. Prior studies in a zebra fish model revealed that a Δ*rpoS* mutant did not exhibit significant attenuation 5 dpi [35]. We monitored the fitness of Δ*rpoS*, Δ*esrB*, and *rpoS*^R99A^ mutants relative to WT *E*. *piscicida* during a 2-week infection in turbot with time series CI analyses (Fig 7D). Consistent with previous PACE-based analyses of genome-wide fitness profiles during chronic *E*. *piscicida* infection of turbot, the Δ*esrB* strain mutant did not show a defect in growth *in vivo* until ~5 dpi and after this point its fitness continued to decline (Fig 7D) [8]. Remarkably, the Δ*rpoS* mutant exhibited the inverse pattern; i.e., it was most attenuated early in infection (at 1–2 dpi), but later, the mutant recovered and by 8–14 dpi it exhibited equal fitness as the WT (Fig 7D). The inverse kinetics of the requirements for *rpoS* and *esrB* support a model where *rpoS* is required early in infection to activate genes required for adaptation to host-derived stresses (e.g. *rpoS*, *cadA1*, *cadB1*, *cadB3*, *uspB*, *cspA*, *cspG*, *cspH*, *cspI*, *speAB*, *speG*, *trxC*, *dps*, *phoR*, *csrA*); later, presumably at the point when the pathogen begins to occupy the niche where T3/T6SS enable growth, *rpoS* becomes dispensable, because its repression of *esrB* inhibits production of these secretion systems. Measurement of RpoS and EseB amounts in liver homogenates from fish infected with WT *E*. *piscicida* generally support the idea the requirement for RpoS wanes during the course of infection. The levels of RpoS peaked at ~8 dpi and then declined, whereas EseB levels peaked on ~11 dpi and remained elevated (S6 Fig). The fitness profile of the *rpoS*^R99A^ strain, which slightly out-competed the WT throughout the 14-days of observation, also supports the idea that repression of *esrB* must be relieved during the course of infection. As shown above, this variant *rpoS* is able to promote expression of genes whose transcription require this sigma factor (Fig 6F), but it does not repress *esrB*. Thus, there may be host signals that lead to inhibition of RpoS expression/activity after *E*. *pisicicida* initially establishes itself within the host environment. Taken together, these data suggest that *E*. *pisicicida* modulates RpoS’ roles promoting expression of stress adaptation genes and repressing virulence gene expression during the course of chronic infection.

## Discussion

**Fig 8:**
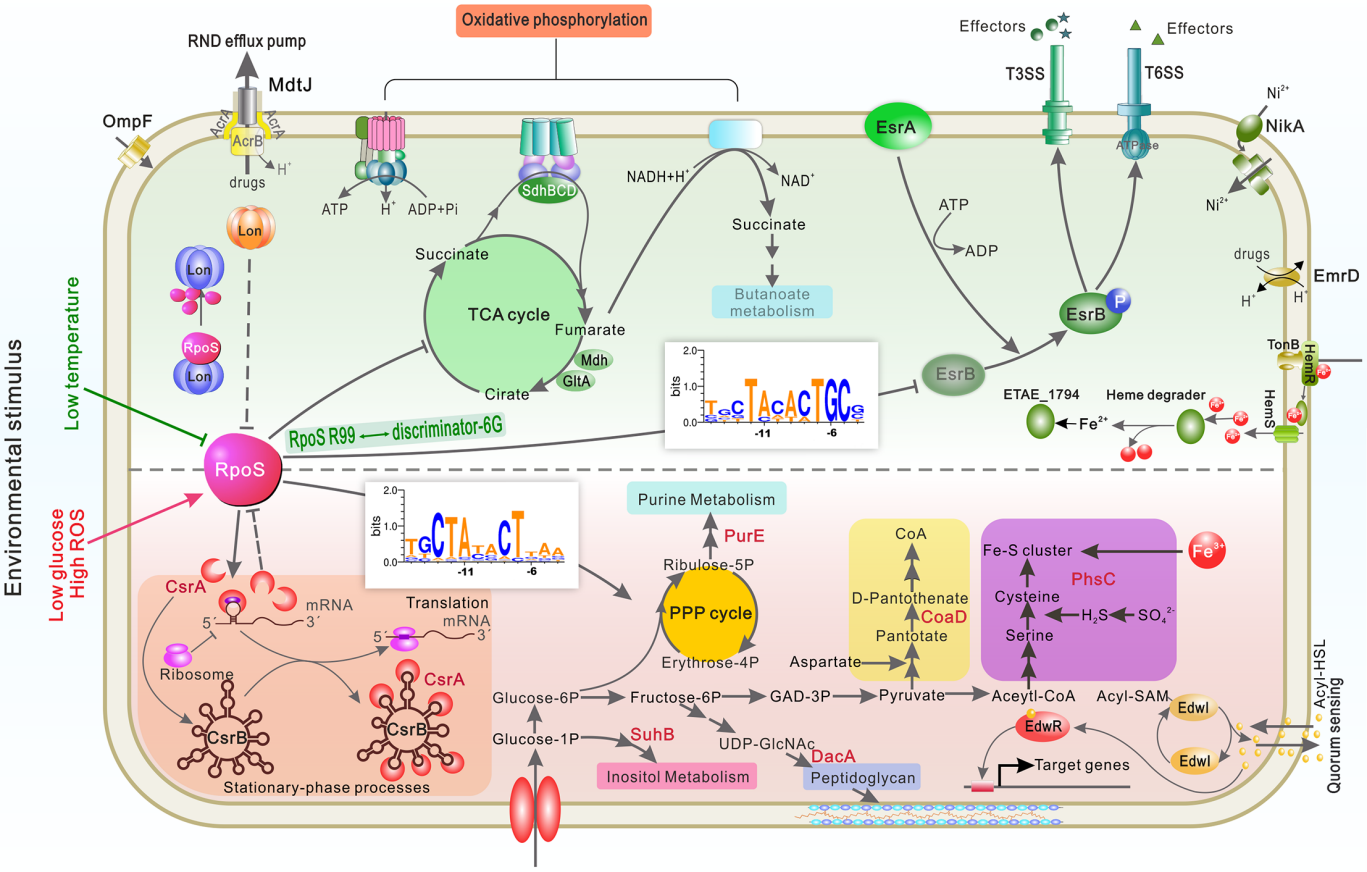
Working model of RpoS control of metabolic and virulence related processes in *E*. *piscicida*. Various stresses including low temperature, high ROS, and starvation modulate the level of RpoS. RpoS bound to core RNAP binds to the -10 and -35 elements in promoters enabling (lower) or blocking (upper) transcription. RpoS interactions with different discriminator sequences promote or impede transcription. Arrowed and bar-ended lines indicate activation and repression, respectively.

Here, we used a genome-wide loss-of-function Tn-seq screen to identify regulators controlling the expression of EsrB, a key activator of *E*. *piscicida* virulence. Unexpectedly, we discovered that RpoS inhibits *esrB* expression, and thus limits production of the pathogen’s T3SS/T6SS. Comparisons of the global transcription profiles of wt and Δ*rpoS* strains showed that RpoS controls expression, directly or indirectly, of more than 700 genes. Several stress stimuli modulate RpoS abundance and thus likely control *esrB* expression and *E*. *piscicida* virulence. Notably, *in vitro* transcription of *esrB* by the RpoD-core RNAP complex (Eσ^70^) was blocked by RpoS. Furthermore, this inhibitory effect, likely mediated by Eσ^38^, was abrogated by mutations in the *esrB* promoter discriminator or by a single amino acid substitution in RpoS R99, a residue in the sigma 1.2 region (the first part of RpoS conserved region 2) that molecular modeling predicted to be in close proximity to the -6G nucleotide of the *esrB* promoter discriminator. Collectively, these observations strongly suggest that direct interactions of Eσ^38^ with the *esrB* promoter impede transcription of this virulence regulator. In a turbot model, RpoS was required for robust *E*. *piscicida* growth during the first few days of infection, whereas EsrB was not; conversely, by 5 dpi RpoS becomes dispensable and EsrB becomes critical. By mediating expression of genes promoting stress responses [35] and inhibiting expression of *esrB*-controlled virulence genes, RpoS activity allows *E*. *piscicida* to co-ordinate expression of diverse cellular pathways (Fig 8). Thus, our findings suggest that the pathogen interprets variations in host-derived signals during the course of infection to modulate RpoS abundance/activity and thereby fine tunes its physiology for growth in different host environments.

RpoS is a key global regulator in many Gram-negative bacteria [20–22, 24]. In *E*. *piscicida*, RpoS was previously shown to be critical for the organism’s adaptation to several stressors, including starvation, high NaCl, H_2_O_2_, as well as serum [35]. Here, using RNA-seq and ChIP-seq, we further refined our knowledge of RpoS control of gene expression in *E*. *piscicida* (S3 Table, S4 Table and S5 Table). Expression of more than 500 genes was upregulated by RpoS while ~200 genes were down regulated by this alternative sigma factor. In general, in *E*. *piscicida* as in other organisms, RpoS promotes expression of genes activated in stationary phase and facilitates stress responses (Fig 8, lower panel; S3 Table) [24]. For example, CsrA, an important RNA chaperone that functions in stationary-phase processes [46], was activated by RpoS. Stress response related genes, including *uspB* and *gadBC* [47–48], were also induced by RpoS. The succinate metabolic pathway (*sdhABCD*), TCA cycle (*citCEFX* and *acnB*) and hemin iron uptake (*hemNPRS*) in *E*. *piscicida* (Fig 8, upper panel; S4 Table) were among the genes most down-regulated by RpoS [22, 24, 35]. The succinate pathway is not only an important step in the tricarboxylic acid (TCA) cycle, but also serves as an electron donor coupled with the oxidative phosphorylation respiratory chain. The repression of the succinate pathway and TCA cycle by RpoS has also been observed in other bacteria, e.g. *Escherichia coli* O157:H7 [26], *Legionella pneumophila* [49] and *S*. *enterica* [29]. Presumably, RpoS represses these metabolic genes via a similar mechanism as its repression of *esrB*; i.e., the RpoS R99 residue directly binds to -6G nucleotide in the discriminators of the respective repressed promoters (Fig 8, upper panel). The mechanism of direct RpoS repression of gene expression uncovered in *E*. *piscicida* may be shared among several Gram-negative bacteria since RpoS repressed genes often contain -6G in their respective promoter discriminators, e.g. in *S*. *enterica* 4 known RpoS repressed genes contain -6G in their respective promoter discriminators (S4B Fig) [29]. RpoS is thought to mediate a trade-off between self-preservation and nutritional competence (SPANC) such as in *S*. *enterica* [49–50]. Our findings suggest that in *E*. *piscicida*, RpoS mediates a different trade-off between stress adaption and virulence; however, it is possible that expression of virulence-associated loci is equivalent to promoting rapid growth (nutritional competence) in certain host environments.

The sigma factor subunit of RNAP holoenzyme enables this multicomponent enzyme to recognize specific promoters during the initiation of transcription [19–21]. Generally, RpoD (σ^70^) mediates recognition of promoters carrying out the cell’s housekeeping function [22–25], while alternative σ factors, like RpoS, mediate transcription of specific subsets of genes in different growth conditions [51–52]. Although sigma factors can enable transcription of repressors or sRNAs that down-regulate expression of target regulons, typically Eσ complexes are not thought to directly block transcription. However, σ factor competition for binding to core RNAP has been thought to explain how one sigma factor can inhibit transcription mediated by another σ factor [28, 53–56]. An alternative means by which Eσ could impede transcription is by binding to and occluding promoter DNA, preventing initiation of transcription. Our findings are consistent with the latter mechanism: Eσ^38^ impairs *esrB* transcription through direct interactions with the *esrB* promoter, particularly with the -6G in the discriminator. Similar Eσ^38^-mediated repression at the level of the *sdh* promoter was also described in *S*. *enterica* serovar Typhimurium [29]. Notably, in this enteric pathogen, as in *E*. *piscicida*, RpoS interactions with the *sdh* promoter discriminator proved critical for repression; thus, when Levi-Meyreuis *et al*. mutated the GCC discriminator in P_*sdh*_ to TAA, RpoS repression was abolished [29]. Our modeling-based mutagenesis of *E*. *piscicida* RpoS extends understanding of the manner in which this alternative sigma factor can block transcription. We show that a particular residue R99 in the sigma 1.2 region is essential for repression but not for Eσ^38^ to initiate transcription. Thus, the manner in which Eσ^38^ interacts with different discriminator sequences appears to determine the outcome of the interaction (preventing or initiating transcription). Analyses of the data garnered from our RNA-seq and ChIP-seq experiments suggests that there are at least 6 RpoS-regulated promoters at which Eσ^38^ directly impairs transcription, suggesting that RpoS control of the cell’s transcriptional output is even more varied and subtle than previously thought.

Many promoters can be simultaneously recognized by RpoD and RpoS, as these sigma factors share similar recognition motifs in their respective -35 and -10 elements [40]. As expected, the -10 element in P_*esrB*_ was critical for *esrB* expression by RpoD or RpoS^R99A^ (Figs 4D and 6C). *In vitro* transcription studies also revealed that in the presence of RpoD, core RNAP, and the wt *esrB* promoter sequence, RpoS inhibited transcription *in vitro*; in the absence of RpoD, Eσ^38^ did not result in transcription from wt P_*esrB*_ but it did from P_*esrB mut2*_ (Fig 4D) and RpoS^R99A^ could drive transcription of P_*esrB*_ and P_*esrB mut2*_ in absence of RpoD as well (Fig 6C). Together, these observations suggest that RpoS interactions with the discriminator modifies the promoter in a manner that renders it resistant to Eσ^70^ binding/initiation. Additional studies, elucidating precisely how RpoS-promoter interactions prevent transcription are warranted.

RpoS has been shown to regulate virulence in several pathogens [57]. In most cases, RpoS is required for virulence. For example, *S*. *enterica* serovar Typhimurium *rpoS* mutants are attenuated, likely because RpoS activates the expression of the plasmid-borne *spvR* and *spvABCD* genes, which are important for intracellular growth [58–59]. RpoS modulation of *E*. *piscicida* pathogenicity is complex and varies during the course of infection. For the first five days of infection, a Δ*rpoS* was attenuated, but after that time the mutant was as fit as the WT (Fig 7D). The bases for the reduced fitness of the Δ*rpoS* mutant requires further definition; however, it is likely that the large set of > 500 genes whose expression is upregulated by RpoS, e.g. stress response genes that promote resistance to host defenses such as H_2_O_2_, facilitate the pathogen’s growth. The early expression of genes ordinarily repressed by RpoS, such as *esrB*, could in principle also account for the attenuation of the Δ*rpoS* mutant. However, this does not seem to be the case, since the strain expressing RpoS^R99A^, which unlike RpoS, does not inhibit *esrB* expression, was not attenuated early in infection.

The inverse kinetics of the requirements for *rpoS* and *esrB* during infection (the *esrB* mutant became attenuated 5 dpi), suggests that the relief of RpoS repression of *esrB*, and production of T3/T6SS, becomes important only several days after the initiation of infection. Consistent with this idea, we found that ectopic expression of *esrB* (from the *lac* promoter) could overcome attenuation caused by over-expression of *rpoS* (Fig 7C). Thus, the level and/or activity of RpoS decreases during the course of infection. Many studies have elucidated the complex cellular factors that govern RpoS levels/activity (reviewed in [22]). Ultimately, environmental conditions, including nutrient availability and stressors, such as hydrogen peroxide, control RpoS activity. Therefore, the relief of RpoS-mediated repression in *esrB* by 5 dpi (reflected in the requirement for EsrB at this point), strongly suggests that *E*. *piscicida* is beginning to occupy a distinct host niche at this point. It will be interesting to further define how the pathogen’s localization, both in terms of host organ and whether it is extra- or intracellular, changes over time. It is tempting to speculate that ~3–5 dpi, when *esrB* becomes critical for *E*. *piscicida* growth, may correspond to the time when the pathogen transitions from growing predominantly extracellularly, in the intestinal lumen and peritoneum, to predominantly intracellularly [7]. It will be fascinating to couple such localization studies with measurements of critical cellular regulators, such as (p)ppGpp, known to control RpoS levels/activity [22], to decipher the molecular factors that govern the activity of this alternative sigma during infection.

## Materials and methods

### Bacterial strains, plasmids, media and antibiotics

The bacterial strains used in this study are listed in S6 Table. *E*. *piscicida* were statically cultured in Luria-Bertani broth (LB) or Dulbecco’s modified essential medium (DMEM) at 28°C, while *Escherichia coli* strains were grown in shaking cultures in LB at 37°C. The *E*. *coli* DH5α λ*pir* strain was used to propagate the *pir*-dependent suicide plasmids and *E*. *coli* SM10 λ*pir* strain was used as the conjugation donor to introduce suicide plasmids into *E*. *piscicida*. *E*. *coli* BL21 (DE3) was used to express recombinant proteins. When required, antibiotics were added at the following concentrations: carbenicillin (Carb, 100 μg/ml), chloramphenicol (Cm, 25 μg/ml), colistin (Col, 16.7 μg/ml), kanamycin (Kan, 100 μg/ml), tetracycline (Tet, 12.5 μg/ml).

### Genetic engineering of *E*. *piscicida*

The construction of in-frame deletion mutants was accomplished using *sacB-*based allelic exchange vectors as previously described [14]. Upstream and downstream fragments were amplified by PCR and then the Gibson assembly method [60] was used to ligate these fragments into the suicide vector pDMK which was linearized with *Xho*I. The vectors were initially propagated in *E*. *coli* DH5α λ*pir* and after sequencing and purification, they were introduced into *E*. *coli* SM10 λ*pir* and subsequently transferred into EIB202 by conjugation. The single crossover strains were selected on LB agar (LBA) medium containing Kan and Col, and the double crossover strains were selected on LBA containing 12% (w/v) L-sucrose as previously described [14].

Vectors for complementation, over-expression, and reporters were constructed with the *E*. *piscicida* compatible and stable plasmid pUTat as previously described [45, 60]. The construction of reporter strains with promoterless luciferase (*luc*), *luxAB* or Kan resistance gene (*kan*) respectively fused to the promoters of *esrB* (P_*esrB*_), *evpA* (P_*evpA*_), *eseB* (P_*eseB*_), *esrB* (P_*esrB*_), and *rpoS* (P_*rpoS*_) and inserted in chromosome were carried out using the same steps employed for generation of in-frame mutants. All the primers used to construct and validate the strains used here are listed in S7 Table.

### Transposon insertion sequencing

The P_*esrB*_*-kan* fusion was inserted into a neutral site (between ETAE_3536-ETAE_3537) [8] in the WT strain. The transposon insertion sequencing was conducted as previously described [8, 61–62]. A modified pSC189 [31], which carried a gene for resistance to tetracycline was used to deliver the Himar transposon; the transposon library was stored in -80°C. Before screening, the library was resuspended in 5 ml of DMEM medium with (Output) or without (Input) addition of kanamycin, and cultured at 28°C for 24 h without shaking. Then the cultures of each group were plated on LBA medium and cultured at 37°C for 12 h. Finally, all of the colonies in each group were collected from the plates and restored in -80°C. The genomic extraction, library construction and sequencing were conducted as previously described [8]. The library was sequenced on the Hiseq 2500 platform (Illumina, San Diego, CA) by GENEWIZ (Suzhou, China). Reads for each output library were normalized based on the input library and the reads per TA site were tallied and assigned to annotated genes or intergenic regions as described [31]. The fold change (FC) and Mann-Whitney U test (MWU) of each locus are based on comparison of the output and input libraries.

### Minimum inhibitory concentration assay

The MIC assay was conducted as previously described [61]. Δ*esrB*::P_*esrB*_*-kan* was constructed as the negative control. A gradient of increasing concentrations of kanamycin were used and all the strains were statically cultured in 96-well plates at 28°C for 24 h. The bacterial growth was also monitored by measuring the optical density at 600 nm (OD_600_) (Biotek, Winooski, VT, USA).

### Fluorescence assay

The fluorescence assays were conducted as previously described [60]. The reporter strains were cultured in 50 ml DMEM medium. Every 3 h, 200 μl were removed from each culture and added to 96-wells plates. The cell densities (OD_600_) were detected with a microplate reader (Biotek, USA) and the fluorescence values were detected with a OrionII Microplate Luminometer (Berthold, Bad Wildbad, Germany).

### Extracellular proteins (ECPs) assay and immunoblot analysis

Bacterial strains were inoculated into LB medium and subcultured in 50 ml DMEM at 28°C for 24 h. After pelleting the cells at 5,000 g for 10 min, protease inhibitors were added to the supernatants which were then filtered through the 0.22 μm low-protein-binding Millex filter (Millipore) and concentrated to 250 μl using a 10-kDa-cutoff Amicon Ultra-15 centrifugal filter device (Millipore). SDS-PAGE was used to detect the ECPs profiles of EIB202 strains as previously described [13].

For the immunoblot analyses, bacterial cell pellets or concentrated ECP were suspended in PBS to normalize the culture densities based on OD_600_ measurements. 20 μl of each normalized sample was loaded onto 12% denaturing polyacrylamide gels. The proteins were resolved using electrophoresis and then transferred to PVDF membranes (Millipore). The membranes were blocked in 10% skim milk powder solution, incubated with a 1:2000 dilution of mouse anti RpoS (Santa cruz), Flag-tag (Beyotime), Lon (Sigma), EseB (GenScript, Nanjing, China), RpoB (OriGene) or DnaK (Huabio, Hangzhou, China) antibodies, and finally incubated with a 1:2000 dilution of anti-mouse peroxidase-conjugated IgG secondary antibodies (Sigma). The ECL reagent (Thermo Fisher) was used to visual the blots.

### Total RNA extraction and qRT-PCR

Overnight cultures of WT and Δ*rpoS* were statically subcultured in DMEM at 28°C for 12 h, respectively. RNA samples were extracted with the RNA isolation kit (Tiangen) as previously described [13] and incubated with DNase I (Promega) for 30 min at 37°C to remove genomic DNA. RNA concentrations were measured with NanoDrop and 1 μg of each sample was used for reverse transcription with PrimeScript II 1st Strand cDNA Synthesis Kit (TaKaRa). The qRT-PCR was conducted on the Applied Biosystems 7500 real-time system (Applied Biosystems, Foster City, CA) in triplicate. The comparative CT (2^-ΔΔCT^) method was used to quantify the relative qualities of each transcript, and the housekeeping *gyrB* gene was used as an internal control. All the primers used are listed in S7 Table.

### RNA-seq

For mRNA-specific RNA-seq, Ribo-Zero-rRNA (Epicentre) was used to remove the rRNA in the RNA samples following the manufacturer’s instructions. The final concentration of RNA samples was determined with the Qubit 2.0 Fluorometer (Thermo Fisher). The VAHTS Stranded mRNA-seq Library Prep Kit for Illumina (VATHS turbo) was used in the construction of strand-specific RNA-seq libraries, and the sequencing was conducted on the Hiseq 2500 platform to yield 100-base-pair end-reads. Adapter sequences and low-quality bases (PHRED quality scores ≤5) were trimmed by the Trimmomatic package using the default parameters, and truncated reads smaller than 35 bp were discarded. The RNA-seq data processing procedures and statistical analysis were the same as previously described [63].

### Pull-down assay

Overnight cultures of Δ*rpoS*/*flag-rpoS* were subcultured in DMEM at 28°C for 24 h. Bacterial pellets were collected and washed using ddH_2_O, and stored at -80°C. After three cycles of treatment at 80°C for 1 h and ice incubation for 1 h, the pellets were resuspended with 3 ml BS/THES buffer (THES Buffer: 50 mmol/L Tris HCl (pH 7.5), 10 mmol/L Sucrose (m/v), 140 mmol/L NaCl, 0.7% Protease Inhibitor Cocktail (v/v); 5× BS Buffer: 50 mmol/L HEPES, 25 mmol/L CaCl_2_, 250 mmol/L KCl, 60% Glycerol; BS/THES Buffer: 44.3% THES Buffer, 20% 5 × BS Buffer, 35.7% ddH2O). Bacteria were cracked by ultrasonication and supernatants were collected after centrifuge. Biotinylated DNA and NeutrAvidin Agarose Resin beads (Thermo Fisher) were mixed and incubated at 25°C for 1 h. Then the probe-labeled beads were washed with TE and BS/THES buffer for two times. The probe-labeled beads and supernatant lysates (containing 200 ng/μl Poly (dI:dC)) were mixed and incubated with slow shaking at 25°C for 30 min. After 4 washes with BS/THES buffer, proteins were eluted from the beads with a NaCl concentration gradient. Finally, the beads were eluted with ddH_2_O under 70°C to unbound the Biotin-DNA. The identification of pull-down samples were the same as previously described [64].

### Purification of His-tagged proteins

Full-length *rpoS* and *rpoD* open reading frames were amplified from EIB202 genomic DNA. The PCR products were subcloned into pET28a and transformed into *E*. *coli* BL21 (DE3). The resulting strains were grown with shaking in LB medium at 37°C until OD_600_ ~ 0.6. Then isopropyl β-D-1-thiogalactopyranoside (IPTG) was added to a final concentration of 0.5 mM, and the cells were cultured at 16°C for another 16 h. The purification procedure was conducted as previously described [13] with the use of HEPES buffers (20 mM HEPES, 250 mM NaCl, x imidazole); the final concentrations of imidazole of binding buffer, washing buffer and elution buffer were 20 mM, 50 mM and 300 mM, respectively. The purified proteins were dialyzed with HG buffer (20 mM HEPES, 250 mM NaCl, 5% glycerol (w/v)) for 20 h to remove the imidazole. The purified proteins were stored at -80°C.

### Chromatin immunoprecipitation sequencing (ChIP-seq)

The pUTat/*flag-rpoS* and pUTat/*flag* plasmids encoding RpoS-Flag and the Flag tag alone, respectively, were expressed in the Δ*rpoS* strain for ChIP assays as previously described [65]. Overnight cultures of each strain were diluted to the same cell density (OD_600_) and statically subcultured in DMEM medium containing carbenicillin at 28°C for 24 h. Then Rifampicin (Sigma) was added at a final concentration of 150 μg/ml, and incubated at 28°C for 30 min. Formaldehyde was used for cross-linking the protein-DNA complexes *in vivo* and the cross-linking was stopped by addition of glycine solution. The ChIP assay was conducted as previously described [65]. The DNA was purified by phenol/chloroform and precipitated with ethanol. For ChIP validation, DNA fragments were PCR amplified with primer pair *esrB*-EF/ER (S7 Table). For ChIP-seq, the DNA fragments were used for library construction with the VAHTS Turbo DNA library prep kit (Vazyme, Nanjing, China), and the number of reads per microliter of each library was determined by qRT-PCR. The sequencing procedure was the same as that described for Tn-seq, and the MEME-suite website (http://meme-suite.org) was used to identify the RpoS binding motif.

### *In vivo* fluorescence detection and competition assays

Healthy turbot fish (average weight of ~30 g) were chosen and acclimatized in the aeration tanks for two weeks with a continuous flow of seawater at 16°C. For *in vivo* fluorescence detection, the subcultures of reporter strains were diluted to 10^6^ CFU/ml in PBS. The fish were anesthetized with tricaine methanesulfonate (MS-222) (Sigma-Aldrich) at a concentration of 80 mg/l. Fish were intraperitoneally (i.p.) injected with 100 μl of bacterial suspensions. At 8 days post injection (dpi), the fish were i.p. injected with beetle luciferin substrate (Promega). After 10 min, the fluorescence was detected with a Kodak In-Vivo Multispectral System FX (Carestream Health). Then the livers of the fish were sampled and bacterial colonization was measured by CFU plating.

For competition assays, inocula were prepared using fresh cultures of bacteria that were diluted and mixed at 1:1 ratio. The injection dose was 10^5^ CFU/fish. At 8 dpi, the livers from fish in each group (5 animals/group) were removed, homogenized and plated on DHL plates with or without containing chloramphenicol (Cm) to enumerate the ratio of the competing strains. The ratios of the bacterial counts were used to determine the CIs.

### Accession numbers

The RNA-seq sequencing data was deposit at SRA (SRP136988).

### Ethics statement

All animal protocols used in this study were approved by the Animal Care Committee of the East China University of Science and Technology (2006272). The Experimental Animal Care and Use Guidelines from Ministry of Science and Technology of China (MOST-2011-02) were strictly followed.

### Originality-significance statement

All the data and related materials are our original research, and have not been previously published and have not been submitted for publication elsewhere while under consideration.

## Supporting information

S1 Fig Determination of appropriate kanamycin concentration for the screen, library saturation and validation of a subset of Tn-seq results. **(A)** Relative growth of WT::P_*esrB*_-*kan* vs its Δ*esrB* derivative in DMEM medium with different amounts of kanamycin. **(B)** Distribution of the percentage of TA site disrupted in the input library. **(C)** qRT-PCR validation of Tn-seq results. The gene disrupted mutants present in a defined EIB202 transposon library created in our lab, i.e. YKY013 (*purA*^-^), YKY014 (*cdsA*^-^), YKY015 (*guaB*^-^), YKY016 (*1412*^-^), YKY017 (*slt*^-^), YKY018 (*mltC*^-^), and YKY019 (*acrB*^-^) were used (S6 Table). The transcript levels of *esrB* were measured with qRT-PCR using the ΔΔC_T_ method. The transcript of *gyrB* was employed as a control.
(TIF)

S2 Fig Flag-tagged RpoS is functional and inhibits cell auto-aggregation. **(A)** The *flag*-*rpoS* was highly expressed in Δ*rpoS* as revealed by western blot of the Flag tag. **(B)** The *flag*-*rpoS* was functional in repression of EseB-mediated cell auto-aggregation phenotype. (**C**) The *flag*-*rpoS* expression levels in the WT, Δ*rpoS*, and *rpoS*^OE^ strains over expression P_*esrB*_-*luxAB*, P_*esr Bmut1*_-*luxAB*, and P_*esr Bmut2*_-*luxAB*.
(TIF)

S3 Fig ChIP-seq analysis of genes directly bound and regulated by RpoS. **(A, a-h)** Illustration of the results of ChIP-seq through peak comparison. The fold enrichment of each of the promoters bound by RpoS is shown. The *gyrB* promoter region is shown as a control. **(B)** The RpoS-binding motif derived from ChIP-seq results and generated by the MEME-suite tool (http://meme-suite.org). The height of each letter represents the relative frequency of each base at each position in the consensus sequence.
(TIF)

S4 Fig Genome-wide analysis of RpoS binding sites and discriminator sequences in RpoS controlled promoters and their association with RpoS activation or repression. **(A-B)** RNA-seq results (S3 Table and S4 Table) were analyzed with the binding motif derived from ChIP-seq with FIMO software (http://meme-suite.org), leading to identification of 16 additional putative genes *in E*. *piscicida* directly regulated by RpoS: 12 activated by RpoS (**A**) and 4 repressed by RpoS (**B**, upper). In addition, 4 RpoS-repressed genes were identified to contain the similar -6G containing discriminator in *S*. *enterica* (**B**, lower) [29]. (**C-D**) Chi-square tests evaluating the significance of the presence -6G -5C sites (**C**) or -6G only (**D**) in RpoS promoters vs. absence of these nucleotides in corresponding sites in RpoS activated or repressed promoters. (**E**) Comparisons of the transcript fold change (derived from RNA-seq) of the RpoS directly controlled genes harboring -6T, -6C, -6A and -6G between WT and Δ*rpoS*. *** *P* < 0.0001 based on ANOVA. (**F-G**) Fluorescence from a promoterless *luxAB* reporter fused to wild type and mutant P_*sdh*_ or P_*1580*_, cloned into plasmid pUTat and introduced into WT or Δ*rpoS* strains after 9 h incubled in DMEM. RpoS levels of the indicated strains were assayed with western blot in **G**. *** *P* < 0.0001 based on student’s *t*-test.
(TIF)

S5 Fig RpoS mutant strains do not exhibit *in vitro* growth defects. *In vitro* competition experiments between the indicated strains were carried in LB medium at 28°C for 24 h.
(TIF)

S6 Fig Immunoblot analyses of EseB and RpoS levels during WT infection. Lysates of livers from 5 infected fish were blotted with anti-EseB and -RpoS specific antisera; RpoB was used as the loading control for the blots. The numbers under the panels correspond to densitometry measurements with the RpoB-level normalized values in brackets. The results shown represent the mean of triplicate experiments and a representative blot is shown. The placement of the triangle separating the 11- and 14- dpi samples was based on the location of the RpoB blots in the same gel.
(TIF)

S1 Table Putative repressors of *esrB* as identified by Tn-seq analysis. (DOCX)

S2 Table Putative activators of *esrB* as identified by Tn-seq analysis. (DOCX)

S3 Table List of genes activated by RpoS according to RNA-seq analysis. (XLSX)

S4 Table List of genes repressed by RpoS according to RNA-seq analysis. (XLSX)

S5 Table Putative binding sites of RpoS as identified by ChIP-seq analysis. (DOCX)

S6 Table The strains and plasmids used in this study. (DOCX)

S7 Table Primers used in this study. (DOCX)
