# Supplementary figures and images for: Critical role for a promoter discriminator in RpoS control of virulence in Edwardsiella piscicida
Source: PLoS Pathog. 2018 Aug 31;14(8):e1007272. doi: 10.1371/journal.ppat.1007272 (PMC6136808; doi:10.1371/journal.ppat.1007272)

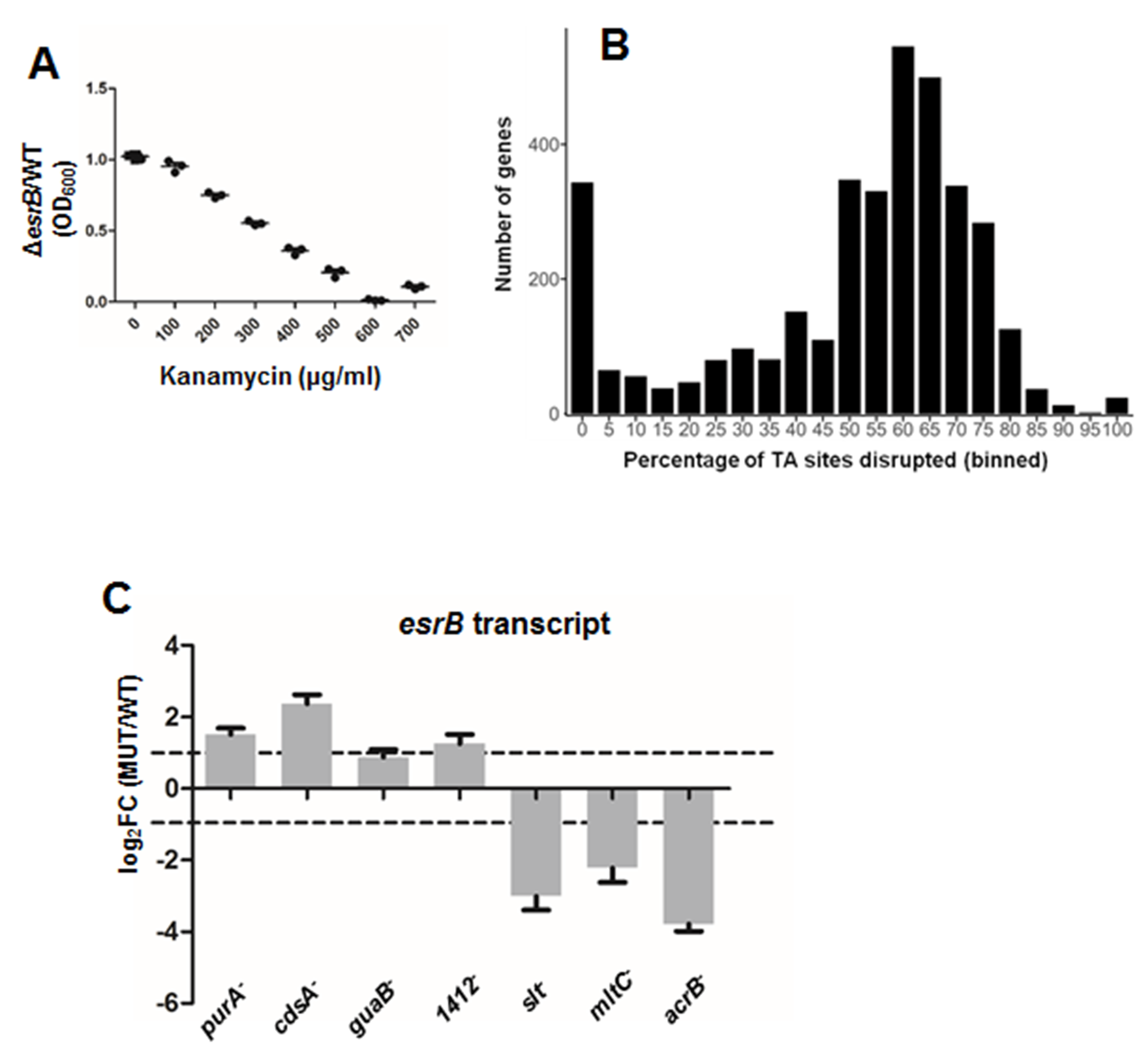

Supplement: S1 Fig — (A) Relative growth of WT::PesrB-kan vs its ΔesrB derivative in DMEM medium with different amounts of kanamycin. (B) Distribution of the percentage of TA site disrupted in the input library. (C) qRT-PCR validation of Tn-seq results. The gene disrupted mutants present in a defined EIB202 transposon library created in our lab, i.e. YKY013 (purA-), YKY014 (cdsA-), YKY015 (guaB-), YKY016 (1412-), YKY017 (slt-), YKY018 (mltC-), and YKY019 (acrB-) were used (S6 Table). The transcript levels of esrB were measured with qRT-PCR using the ΔΔCT method. The transcript of gyrB was employed as a control. (TIF) [file ppat.1007272.s001.tif]

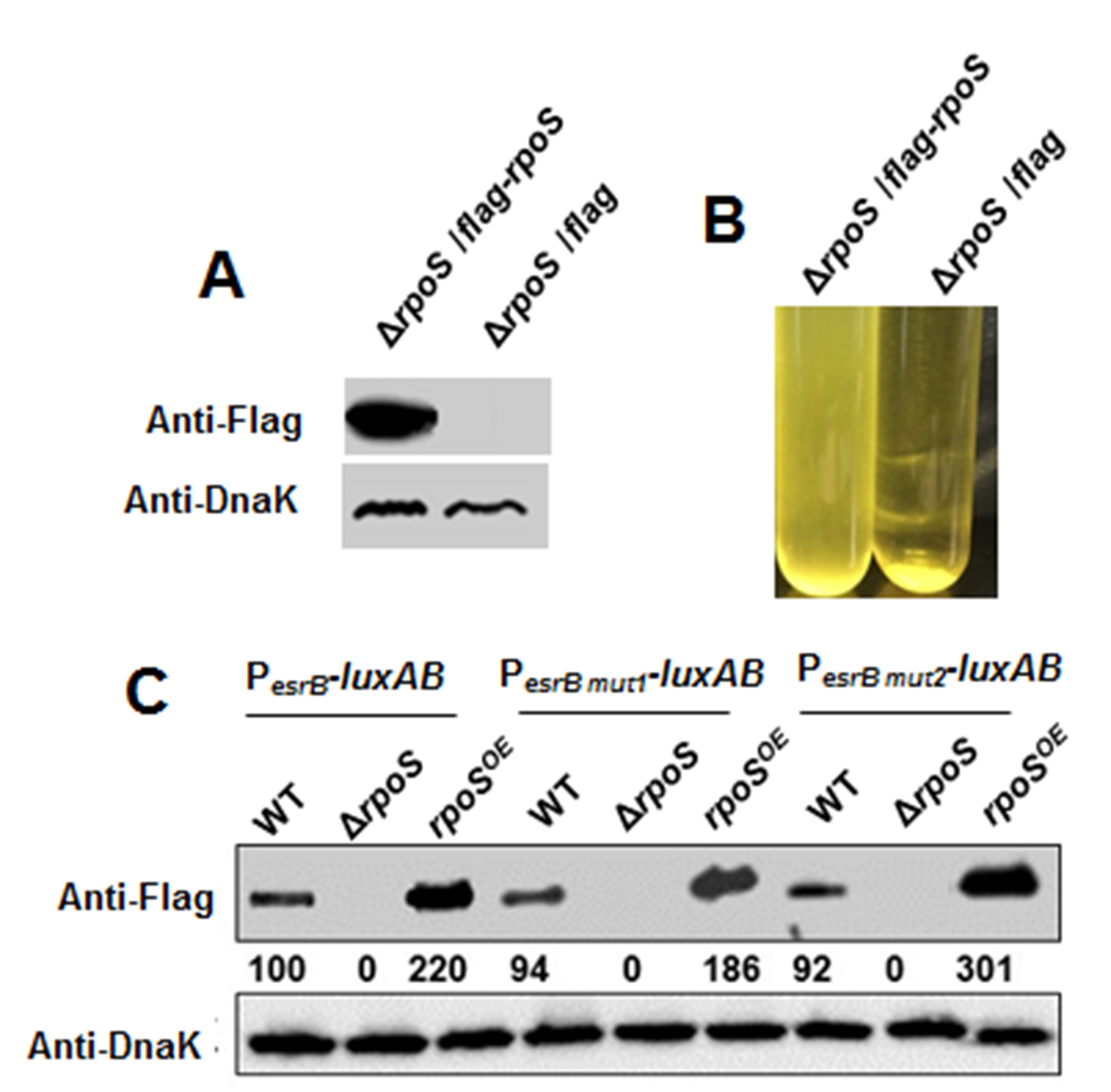

Supplement: S2 Fig — (A) The flag-rpoS was highly expressed in ΔrpoS as revealed by western blot of the Flag tag. (B) The flag-rpoS was functional in repression of EseB-mediated cell auto-aggregation phenotype. (C) The flag-rpoS expression levels in the WT, ΔrpoS, and rpoSOE strains over expression PesrB-luxAB, Pesr Bmut1-luxAB, and Pesr Bmut2-luxAB. (TIF) [file ppat.1007272.s002.tif]

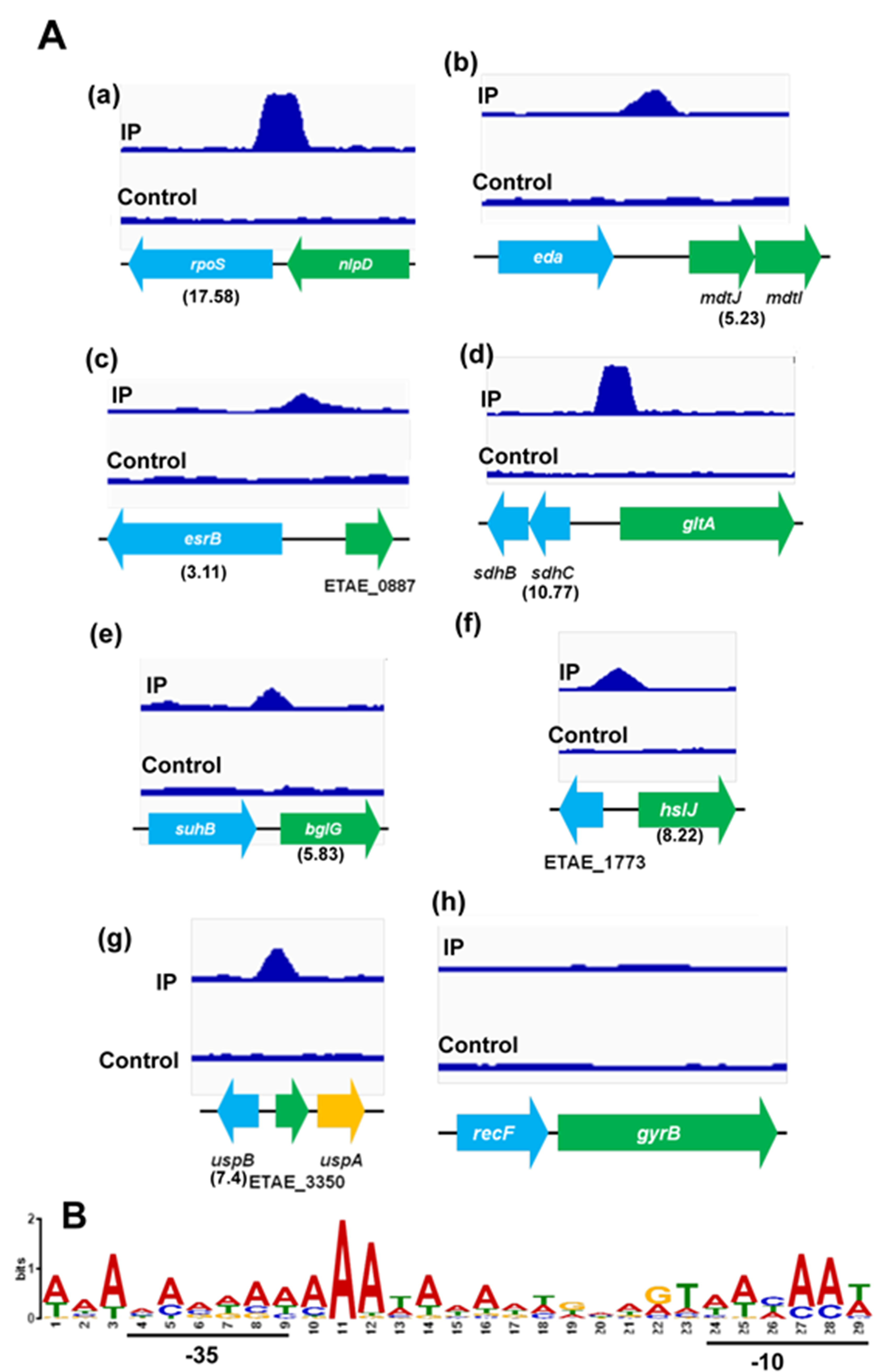

Supplement: S3 Fig — (A, a-h) Illustration of the results of ChIP-seq through peak comparison. The fold enrichment of each of the promoters bound by RpoS is shown. The gyrB promoter region is shown as a control. (B) The RpoS-binding motif derived from ChIP-seq results and generated by the MEME-suite tool (http://meme-suite.org). The height of each letter represents the relative frequency of each base at each position in the consensus sequence. (TIF) [file ppat.1007272.s003.tif]

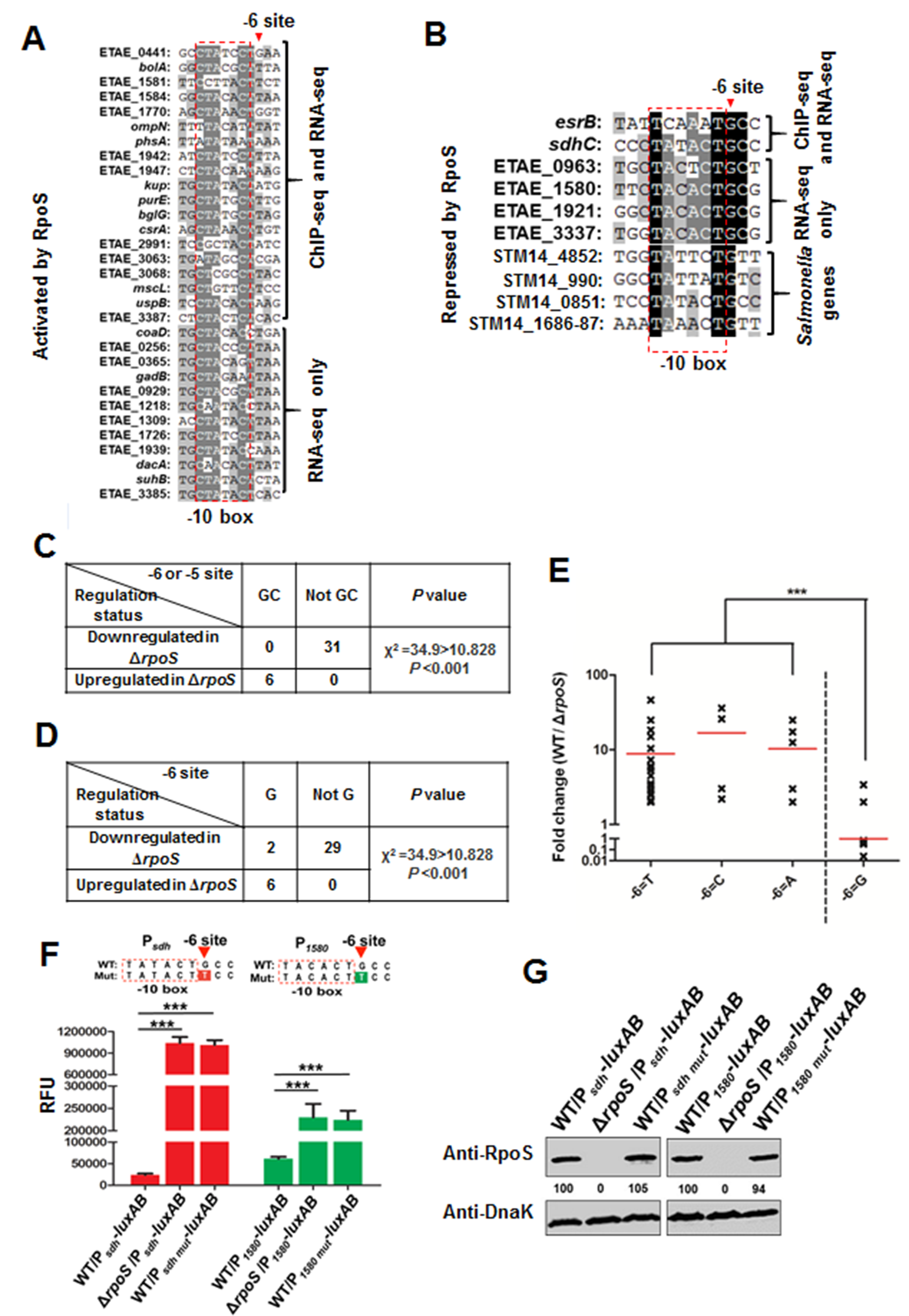

Supplement: S4 Fig — (A-B) RNA-seq results (S3 Table and S4 Table) were analyzed with the binding motif derived from ChIP-seq with FIMO software (http://meme-suite.org), leading to identification of 16 additional putative genes in E. piscicida directly regulated by RpoS: 12 activated by RpoS (A) and 4 repressed by RpoS (B, upper). In addition, 4 RpoS-repressed genes were identified to contain the similar -6G containing discriminator in S. enterica (B, lower) [29]. (C-D) Chi-square tests evaluating the significance of the presence -6G -5C sites (C) or -6G only (D) in RpoS promoters vs. absence of these nucleotides in corresponding sites in RpoS activated or repressed promoters. (E) Comparisons of the transcript fold change (derived from RNA-seq) of the RpoS directly controlled genes harboring -6T, -6C, -6A and -6G between WT and ΔrpoS. *** P < 0.0001 based on ANOVA. (F-G) Fluorescence from a promoterless luxAB reporter fused to wild type and mutant Psdh or P1580, cloned into plasmid pUTat and introduced into WT or ΔrpoS strains after 9 h incubled in DMEM. RpoS levels of the indicated strains were assayed with western blot in G. *** P < 0.0001 based on student’s t-test. (TIF) [file ppat.1007272.s004.tif]

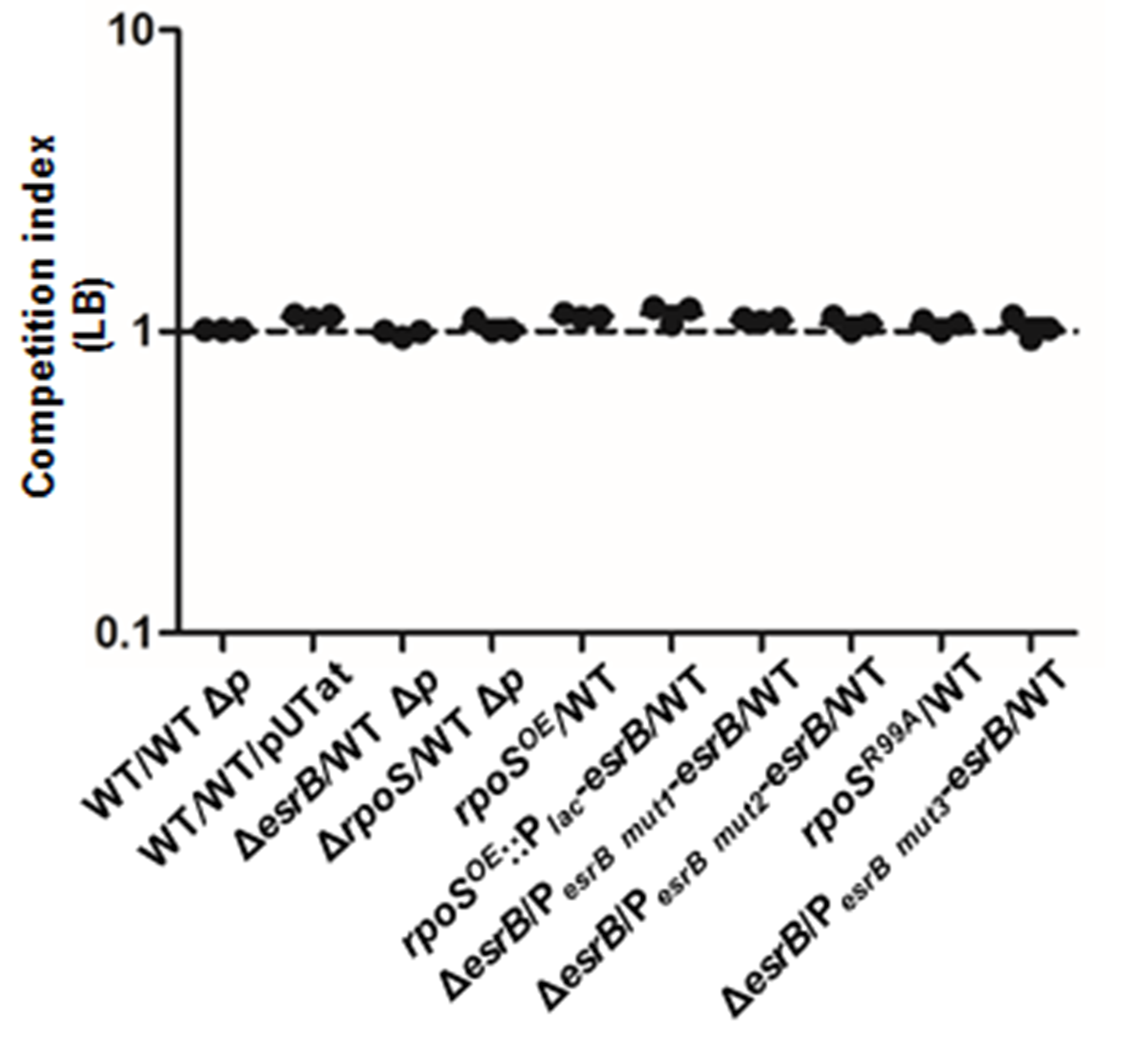

Supplement: S5 Fig — In vitro competition experiments between the indicated strains were carried in LB medium at 28°C for 24 h. (TIF) [file ppat.1007272.s005.tif]

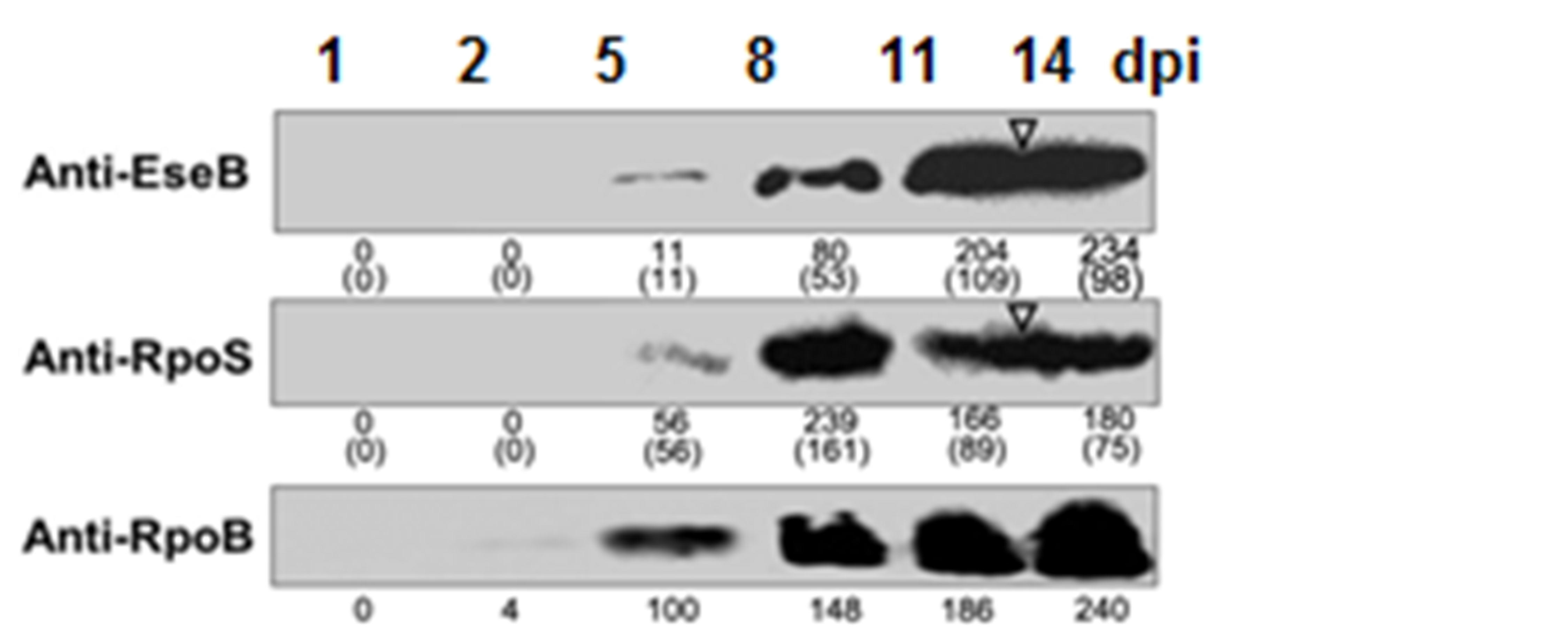

Supplement: S6 Fig — Lysates of livers from 5 infected fish were blotted with anti-EseB and -RpoS specific antisera; RpoB was used as the loading control for the blots. The numbers under the panels correspond to densitometry measurements with the RpoB-level normalized values in brackets. The results shown represent the mean of triplicate experiments and a representative blot is shown. The placement of the triangle separating the 11- and 14- dpi samples was based on the location of the RpoB blots in the same gel. (TIF) [file ppat.1007272.s006.tif]
