# Supplementary material for: Critical role for a promoter discriminator in RpoS control of virulence in Edwardsiella piscicida
Source: PLoS Pathog. 2018 Aug 31;14(8):e1007272. doi: 10.1371/journal.ppat.1007272 (PMC6136808; doi:10.1371/journal.ppat.1007272)
Supplement: S1 Table — (DOCX) [file ppat.1007272.s007.docx]

**Table S1 Putative repressors of *esrB* as identified by Tn-seq**

| **CDS** | **Gene** | **Annotation** | **Fold**  **(Output/Input)** | ***p* value** |
| --- | --- | --- | --- | --- |
| ETAE_0749 | *lpxA* | UDP-N-acetylglucosamine acyltransferase | ∞ | 0.014179 |
| ETAE_3561 | *rpsK* | ribosomal subunit protein S11 | ∞ | 0.045869 |
| ETAE_0035 |  | putative Zn peptidase | 10.56 | 0.011381 |
| ETAE_2873 | *rpoS* | RNA polymerase sigma factor | 7.95 | 0.041361 |
| ETAE_0154 | *hemB* | delta-aminolevulinic acid dehydratase | 4.56 | 0.003064 |
| ETAE_0743 | *cdsA* | CDP-diglyceride synthetase | 3.96 | 0.028112 |
| ETAE_2082 | *pyrF* | OMP decarboxylase; OMPDCase; OMPdecase | 3.53 | 0.047763 |
| ETAE_1096 | *dapA* | dihydrodipicolinate synthase | 3.14 | 0.013304 |
| ETAE_2697 | *cysS* | cysteinyl-tRNA synthetase | 2.92 | 0.022471 |
| ETAE_1760 |  | IS629 orfA | 2.48 | 0.040753 |
| ETAE_1837 |  | hypothetical protein | 2.37 | 0.007016 |
| ETAE_1202 | *ugd* | UDP-glucose 6-dehydrogenase | 2.36 | 0.022232 |
| ETAE_2640 | *holA* | DNA polymerase III subunit delta | 2.36 | 0.004506 |
| ETAE_1439 | *ruvA* | holliday junction resolvasome, DNA-binding subunit | 2.34 | 0.036119 |
| ETAE_0358 | *purA* | adenylosuccinate synthase | 2.23 | 0.006692 |
| ETAE_2813 | *iscA* | iron-sulfur cluster assembly protein | 2.23 | 0.030817 |
| ETAE_2787 | *guaB* | inositol-5-monophosphate dehydrogenase | 2.22 | 0.013466 |
| ETAE_1412 |  | hypothetical protein | 2.10 | 0.013701 |
| ETAE_0430 |  | putative membrane-associated protein | 2.08 | 0.00723 |
| ETAE_0659 | *aceE* | pyruvate dehydrogenase subunit E1 | 2.04 | 0.016385 |
| ETAE_2773 | *mtnN* | 5'-methylthioadenosine/S-adenosylhomocysteine nucleosidase | 2.03 | 0.027142 |
| ETAE_2468 |  | hypothetical protein | 2.02 | 0.034098 |
| ETAE_1614 |  | regulatory prophage protein cI | 2.02 | 0.005862 |
