# Supplementary material for: Critical role for a promoter discriminator in RpoS control of virulence in Edwardsiella piscicida
Source: PLoS Pathog. 2018 Aug 31;14(8):e1007272. doi: 10.1371/journal.ppat.1007272 (PMC6136808; doi:10.1371/journal.ppat.1007272)
Supplement: S2 Table — (DOCX) [file ppat.1007272.s008.docx]

**Table S2 Putative activators of *esrB* as identified by Tn-seq**

| **CDS** | **Gene** | **Annotation** | **Fold**  **(Output/Input)** | ***p* value** |
| --- | --- | --- | --- | --- |
| ETAE_3542 | phoU | phosphate uptake regulator | 0.49 | 0.031469 |
| ETAE_3449 | tpiA | Triosephosphate isomerase | 0.43 | 0.029668 |
| ETAE_1655 | prc | carboxy-terminal protease | 0.42 | 0.049195 |
| ETAE_1158 |  | hypothetical protein | 0.41 | 0.010394 |
| ETAE_0772 | tadA | tRNA-specific adenosine deaminase | 0.40 | 0.03665 |
| ETAE_0695 | mrcB | penicillin-binding protein 1b | 0.37 | 0.001162 |
| ETAE_1170 |  | hypothetical protein | 0.37 | 0.048475 |
| ETAE_1562 |  | hypothetical protein | 0.32 | 0.039546 |
| ETAE_3453 | cpxR | response regulators consisting of a CheY-like receiver domain and a winged-helix DNA-binding domain | 0.32 | 0.004694 |
| ETAE_0263 | mltC | murein transglycosylase C | 0.19 | 0.004885 |
| ETAE_0542 | slt | lytic murein transglycosylase | 0.19 | 0.000331 |
| ETAE_0994 | lon | ATP-dependent Lon protease, bacterial type | 0.16 | 0.005682 |
| ETAE_1425 | mviN | integral membrane protein | 0.14 | 0.008336 |
| ETAE_2638 | leuS | leucyl-tRNA synthetase | 0.11 | 0.015722 |
| ETAE_3207 | rplQ | 50S ribosomal protein L17 | 0.09 | 0.019603 |
| ETAE_1010 | acrB | RND family, acridine/multidrug efflux pump/acriflavin resistance protein B | 0.09 | 0.031747 |
