# Supplementary material for: Critical role for a promoter discriminator in RpoS control of virulence in Edwardsiella piscicida
Source: PLoS Pathog. 2018 Aug 31;14(8):e1007272. doi: 10.1371/journal.ppat.1007272 (PMC6136808; doi:10.1371/journal.ppat.1007272)
Supplement: S5 Table — (DOCX) [file ppat.1007272.s011.docx]

**Table S5 Putative binding sites of *esrB* as identified by ChIP-seq**

| **Peaks** | **Putative CDS** | **Strand** | **Loc-s** | **Loc-e** | **Annotation** | **RNA-seq*** | **Fold_enrichment** |
| --- | --- | --- | --- | --- | --- | --- | --- |
| 49198 | ETAE_0051 | + | 49958 | 50074 | hypothetical protein | NS | 5.8 |
| 410994 | ETAE_0366 | + | 411207 | 411602 | 30S ribosomal protein S6 | NS | 5.41 |
| 486377 | ETAE_0442 | + | 486074 | 486982 | hypothetical transport protein | + | 4.97 |
| 493287 | ETAE_0448 | - | 492511 | 493170 | DNA-binding response regulator in two-component regulatory system with QseC | NS | 4.33 |
| 935393 | ETAE_0850 | + | 935798 | 936397 | putative transmembrane hydrogenase cytochrome B-type subunit | NS | 4.33 |
| 965476 | ETAE_0886 | - | 964658 | 965302 | two-component sensor/regulator | - | 3.11 |
| 1080181 | ETAE_0989 | + | 1080312 | 1080638 | transcriptional regulator | + | 5.35 |
| 1099118 | ETAE_1009 | - | 1098573 | 1098941 | hypothetical protein | NS | 6.12 |
| 1109585 | ETAE_1017 | - | 1109023 | 1109562 | primosomal replication priB and priC | NS | 5.63 |
| 1233651 | ETAE_1136 | + | 1233670 | 1234635 | cell division protein | NS | 5.19 |
| 1243225 | ETAE_1142 | + | 1243278 | 1244516 | manganese transport protein | NS | 6.18 |
| 1283286 | ETAE_1183 | + | 1283554 | 1283994 | UspA domain protein | NS | 6.04 |
| 1362791 | ETAE_1252 | - | 1361552 | 1362106 | putative pilin protein | NS | 6.08 |
| 1510947 | ETAE_1399 | - | 1510079 | 1510669 | peptidyl-tRNA hydrolase | NS | 6.28 |
| 1566438 | ETAE_1453 | + | 1566817 | 1567200 | small multidrug resistance protein | NS | 5.23 |
| 1689309 | ETAE_1581 | + | 1689361 | 1689726 | hypothetical protein | + | 5.05 |
| 1692746 | ETAE_1584 | - | 1692260 | 1692694 | hypothetical protein | + | 6.32 |
| 1700062 | ETAE_1592 | - | 1699518 | 1699856 | pseudogene | NS | 5.04 |
| 1726906 | ETAE_1625 | + | 1727399 | 1727992 | hypothetical protein | NS | 7.54 |
| 1741174 | ETAE_1639 | + | 1742286 | 1742843 | putative virulence-related membrane protein | NS | 5.2 |
| 1882897 | ETAE_1770 | - | 1880166 | 1882769 | hypothetical protein | + | 3.38 |
| 1884438 | ETAE_1774 | + | 1884535 | 1884954 | heat shock protein HslJ | NS | 8.22 |
| 1935767 | ETAE_1826 | + | 1936022 | 1937305 | outer membrane protein | + | 4.43 |
| 1955535 | ETAE_1845 | - | 1953036 | 1955315 | thiosulfate reductase precursor | + | 5.54 |
| 2051254 | ETAE_1943 | + | 2051414 | 2051767 | putative phage glucose translocase | + | 9.29 |
| 2054644 | ETAE_1947 | + | 2055013 | 2055456 | putative acetyltransferase | + | 5.53 |
| 2239443 | ETAE_2123 | - | 2239077 | 2239235 | hypothetical protein | NS | 4.62 |
| 2342764 | ETAE_2222 | + | 2342984 | 2344474 | NAD-dependent epimerase/dehydratase | NS | 4.7 |
| 2390487 | ETAE_2272 | - | 2390005 | 2390154 | hypothetical protein | NS | 4.73 |
| 2445254 | ETAE_2328 | + | 2445301 | 2446020 | transcriptional regulator, TetR family | NS | 4.26 |
| 2598587 | ETAE_2467 | - | 2596267 | 2598423 | putative tail fiber protein | NS | 3.69 |
| 2720110 | ETAE_2588 | - | 2719501 | 2719890 | succinate dehydrogenase cytochrome b556 large membrane subunit | - | 10.77 |
| 2816735 | ETAE_2681 | + | 2816766 | 2818646 | KUP system potassium uptake protein | + | 4.66 |
| 2835471 | ETAE_2700 | + | 2835673 | 2836182 | phosphoribosylaminoimidazole carboxylase catalytic subunit | + | 7.8 |
| 2887389 | ETAE_2749 | + | 2886849 | 2887085 | hypothetical protein | NS | 4.73 |
| 2893088 | ETAE_2758 | + | 2893350 | 2894075 | putative ribonuclease, T2 family | NS | 3.63 |
| 2956736 | ETAE_2820 | + | 2956828 | 2957859 | transcriptional antiterminator | + | 5.83 |
| 3002449 | ETAE_2858 | - | 3002127 | 3002312 | carbon storage regulator | + | 6.32 |
| 3025456 | ETAE_2873 | - | 3025094 | 3026080 | RNA polymerase sigma factor | + | 17.58 |
| 3108695 | ETAE_2946 | - | 3107811 | 3108383 | hypothetical protein | NS | 3.59 |
| 3135150 | ETAE_2972 | - | 3134618 | 3135082 | guanine-specific ribonuclease N1 and T1 | NS | 5.4 |
| 3153767 | ETAE_2991 | + | 3154115 | 3155119 | hypothetical protein | + | 5.07 |
| 3195887 | ETAE_3031 | - | 3194847 | 3195572 | haloacid dehalogenase, type II | NS | 2.78 |
| 3204016 | ETAE_3041 | + | 3204034 | 3204789 | hypothetical protein | NS | 4.46 |
| 3225435 | ETAE_3063 | + | 3225793 | 3226404 | hypothetical protein | + | 6.13 |
| 3229484 | ETAE_3068 | + | 3229764 | 3230003 | hypothetical protein | + | 3.39 |
| 3245947 | ETAE_3083 | + | 3245980 | 3247689 | putative transposase | NS | 2.51 |
| 3265441 | ETAE_3102 | + | 3265542 | 3267281 | sulfonate/nitrate/taurine transport system permease protein | NS | 5.64 |
| 3356024 | ETAE_3184 | + | 3356188 | 3359019 | excinuclease ABC, A subunit | NS | 4.05 |
| 3359883 | ETAE_3186 | - | 3359443 | 3359871 | hypothetical protein | NS | 4.41 |
| 3388783 | ETAE_3204 | + | 3388796 | 3389203 | large-conductance mechanosensitive channel | + | 4 |
| 3455820 | ETAE_3283 | + | 3456046 | 3456297 | ferrous iron transport protein A | NS | 5.13 |
| 3533880 | ETAE_3349 | - | 3533401 | 3533736 | universal stress protein | + | 7.4 |
| 3578391 | ETAE_3387 | + | 3578530 | 3580095 | hypothetical protein | + | 6.15 |
| 3599802 | ETAE_3405 | + | 3599898 | 3600044 | hypothetical protein | NS | 2.5 |
| 3602684 | ETAE_3410 | + | 3602681 | 3602800 | hypothetical protein | NS | 6.97 |
| 3639426 | ETAE_3450 | - | 3638278 | 3639240 | 6-phosphofructokinase | NS | 3.9 |

*: “+” the genes down-regulated in Δ*rpoS*, “-” the genes upregulated in Δ*rpoS*, “NS” genes had no significant changes in Δ*rpoS.*
