# Supplementary material for: Critical role for a promoter discriminator in RpoS control of virulence in Edwardsiella piscicida
Source: PLoS Pathog. 2018 Aug 31;14(8):e1007272. doi: 10.1371/journal.ppat.1007272 (PMC6136808; doi:10.1371/journal.ppat.1007272)
Supplement: S6 Table — (DOCX) [file ppat.1007272.s012.docx]

**Table S6 The strains and plasmids used in this study**

| **Strains or plasmids** | **Description** | **References** | |
| --- | --- | --- | --- |
| ***Edwardsiella piscicida*** |  |  | |
| YKY001 (EIB202 WT) | Wild-type strain, CCTCC M208068, Col^r^, Str^r^, Cm^r^ | [14] | |
| YKY002 (Δ*esrB*) | EIB202, in-frame deletion of *esrB*, Col^r^_,_ Str^r^, Cm^r^ | [14] | |
| YKY003 (Δ*rpoS*) | EIB202, in-frame deletion of *rpoS*, Col^r^_,_ Str^r^, Cm^r^ | This study | |
| YKY004 (EIB202 ΔP) | EIB202, pEIB202 cured, Col^r^ | [13] | |
| YKY005 (WT::P*_esrB_-kan*) | EIB202, P*_esrB_-kan* in the neutral position, containing pUTat, Col^r^, Str^r^, Cm^r^, Amp^r^ | This study | |
| YKY006 (WT/pUTat) | Wild-type containing pUTat, Col^r^, Str^r^, Cm^r^, Amp^r^ | This study | |
| YKY007 (Δ*rpoS*/pUTat) | Δ*rpoS* containing pUTat, Col^r^, Str^r^, Cm^r^, Amp^r^ | This study | |
| YKY008 (Δ*esrB*/pUTat) | Δ*esrB* containing pUTat, Col^r^, Str^r^, Cm^r^, Amp^r^ | This study | |
| YKY009 (*rpoS*^+^) | Δ*rpoS* containing pUTat-*rpoS*, Col^r^, Str^r^, Cm^r^, Amp^r^ | This study | |
| YKY010 (*rpoS^OE^*) | Δ*rpoS* containing pUTat-P*_rpsU_*-*rpoS*, Col^r^, Str^r^, Cm^r^_,_ Amp^r^ | This study | |
| YKY011 (Δ*esrB*Δ*rpoS*) | EIB202, in-frame deletion of *esrB* and *rpoS*, Col^r^_,_ Str^r^, Cm^r^ | This study | |
| YKY012 (Δ*esrB rpoS*^OE^) | Δ*esrB*Δ*rpoS* containing pUTat-P*_rpsU_*-*rpoS*, Col^r^, Str^r^, Cm^r^_,_ Amp^r^ | This study | |
| YKY013 (*purA*^-^) | Transposon insertion mutant at 1117 site in the *purA orf*, Col^r^, Gm^r^ | Lab collection | |
| YKY014 (*cdsA*^-^) | Transposon insertion mutant at 856 site in the *cdsA orf*, Col^r^, Gm^r^ | Lab collection | |
| YKY015 (*guaB*^-^) | Transposon insertion mutant at 129 site in the *guaB orf*, Col^r^, Gm^r^ | Lab collection | |
| YKY016 (1412^-^) | Transposon insertion mutant at 57 site in the ETAE_1412 *orf*, Col^r^, Gm^r^ | Lab collection | |
| YKY017 (*slt*^-^) | Transposon insertion mutant at 810 site in the *slt orf*, Col^r^, Gm^r^ | Lab collection | |
| YKY018 (*mltC*^-^) | Transposon insertion mutant at 466 site in the *mltC orf*, Col^r^, Gm^r^ | Lab collection | |
| YKY019 (*acrB*^-^) | Transposon insertion mutant at 1965 site in the *acrB orf*, Col^r^, Gm^r^ | Lab collection | |
| YKY020 (Δ*esrB*::P*_esrB_-kan*) | Δ*esrB*, P*_esrB_-kan* in the neutral position, containing pUTat, Col^r^, Str^r^, Cm^r^, Amp^r^ | This study | |
| YKY021 (Δ*rpoS*::P*_esrB_-kan*) | Δ*rpoS*, P*_esrB_-kan* in the neutral position, containing pUTat, Col^r^, Str^r^, Cm^r^, Amp^r^ | This study | |
| YKY022 (*rpoS*^+^::P*_esrB_-kan*) | *rpoS^+^*, P*_esrB_-kan* in the neutral position, containing pUTat-*rpoS*, Col^r^, Str^r^, Cm^r^, Amp^r^ | This study | |
| YKY023 (WT::P*_esrB_*-*luxAB*) | EIB202, P*_esrB_-kan* in the neutral position, containing pUTat, Col^r^, Str^r^, Cm^r^_,_ Amp^r^ | This study | |
| YKY024 (Δ*rpoS*::P*_esrB_-luxAB*) | Δ*rpoS*, P*_esrB_-kan* in the neutral position, containing pUTat, Col^r^, Str^r^, Cm^r^_,_ Amp^r^ | This study | |
| YKY025 (*rpoS*^+^::P*_esrB_-luxAB*) | *rpoS^+^*, P*_esrB_-kan* in the neutral position, Col^r^, Str^r^, Cm^r^_,_ Amp^r^ | This study | |
| YKY026 (*rpoS*^OE^::P*_esrB_-luxAB*) | *rpoS*^OE^, P*_esrB_-kan* in the neutral position, Col^r^, Str^r^, Cm^r^_,_ Amp^r^ | This study | |
| YKY027 (*lon^OE^*) | Wild-type containing pUTat-*lon*, Col^r^, Str^r^, Cm^r^_,_ Amp^r^ | This study | |
| YKY028 (*lon^OE^*::P*_esrB_-luxAB*) | *lon^OE^*, P*_esrB_-luxAB* in a neutral position, Col^r^, Str^r^, Cm^r^_,_ Amp^r^ | This study | |
| YKY029 (Δ*rpoS/flag*) | Δ*rpoS* containing pUTat-P*_rpsU_*-*flag*, Col^r^, Str^r^, Cm^r^_,_ Amp^r^ | This study | |
| YKY030 (Δ*rpoS*/*flag-rpoS*) | Δ*rpoS* containing pUTat-P*_rpsU_*-*flag-rpoS*, Col^r^, Str^r^, Cm^r^_,_ Amp^r^ | This study | |
| YKY031 (WT::P*_esrB mut 1_-luxAB*) | Wild-type, P*_esrB mut 1_-luxAB* in a neutral position, containing pUTat, Col^r^, Str^r^, Cm^r^, Amp^r^ | This study | |
| YKY032 (Δ*rpoS::*P*_esrB mut 1_-luxAB*) | Δ*rpoS*, P*_esrB mut 1_-luxAB* in a neutral position, containing pUTat, Col^r^, Str^r^, Cm^r^, Amp^r^ | This study | |
| YKY033 (WT:: P*_esrB mut 2_-luxAB*) | Wild-type, P*_esrB mut 2_-luxAB* in a neutral position, containing pUTat, Col^r^, Str^r^, Cm^r^, Amp^r^ | This study | |
| YKY034 (Δ*rpoS::*P*_esrB mut 2_-luxAB*) | Δ*rpoS*, P*_esrB mut 2_-luxAB* in a neutral position, containing pUTat, Col^r^, Str^r^, Cm^r^, Amp^r^ | This study | |
| YKY037 (WT/P*_esrB1_-luxAB*) | Wild-type containing pUTat-P*_esrB1_-luxAB*, Col^r^, Str^r^, Cm^r^, Amp^r^ | This study | |
| YKY038 (WT/P*_esrB2_-luxAB*) | Wild-type containing pUTat-P*_esrB2_-luxAB*, Col^r^, Str^r^, Cm^r^, Amp^r^ | This study | |
| YKY039 (WT/P*_esrB3_-luxAB*) | Wild-type containing pUTat-P*_esrB3_-luxAB*, Col^r^, Str^r^, Cm^r^, Amp^r^ | This study | |
| YKY040 (WT/P*_esrB4_-luxAB*) | Wild-type containing pUTat-P*_esrB4_-luxAB*, Col^r^, Str^r^, Cm^r^, Amp^r^ | This study | |
| YKY041 (WT/P*_esrB5_*-*luxAB*) | Wild-type containing pUTat-P*_esrB5_*-*luxAB*, Col^r^, Str^r^, Cm^r^, Amp^r^ | This study | |
| YKY042 (WT/P_esrB6_-*luxAB*) | Wild-type containing pUTat-P*_esrB6_*-*luxAB*, Col^r^, Str^r^, Cm^r^, Amp^r^ | This study | |
| YKY043 (WT/P_esrB7_-*luxAB*) | Wild-type containing pUTat-P*_esrB7_*-*luxAB*, Col^r^, Str^r^, Cm^r^, Amp^r^ | This study | |
| YKY044 (WT/P_esrB8_-*luxAB*) | Wild-type containing pUTat-P*_esrB8_*-*luxAB*, Col^r^, Str^r^, Cm^r^, Amp^r^ | This study | |
| YKY045 (WT/P_esrB9_-*luxAB*) | Wild-type containing pUTat-P*_esrB9_*-*luxAB*, Col^r^, Str^r^, Cm^r^, Amp^r^ | This study | |
| YKY046 (*rpoS^L61A^*) | Δ*rpoS* containing pUTat-P*_rpsU_*-*rpoS^L61A^*, Col^r^, Str^r^, Cm^r^, Amp^r^ | This study | |
| YKY047 (*rpoS^R99A^*) | Δ*rpoS* containing pUTat-P*_rpsU_*-*rpoS*^R99A^, Col^r^, Str^r^, Cm^r^, Amp^r^ | This study | |
| YKY048 (*rpoS^L61AR99A^*) | Δ*rpoS* containing pUTat-P*_rpsU_*-*rpoS*^L61AR99A^, Col^r^, Str^r^, Cm^r^, Amp^r^ | This study | |
| YKY049 (WT*/*P*_sdh_-luxAB*) | Wild-type containing pUTat-P*_sdh_*-*luxAB*, Col^r^, Str^r^, Cm^r^, Amp^r^ | This study | |
| YKY050 (Δ*rpoS/P_sdh_-luxAB*) | Δ*rpoS* containing pUTat-P*_sdh_*-*luxAB*, Col^r^, Str^r^, Cm^r^, Amp^r^ | This study | |
| YKY051 (WT*/*P*_sdh mut_-luxAB*) | Wild-type containing pUTat-P*_sdh mut_*-*luxAB*, Col^r^, Str^r^, Cm^r^, Amp^r^ | This study | |
| YKY052 (WT*/P_1580_-luxAB*) | Wild-type containing pUTat-P*_1580_*-*luxAB*, Col^r^, Str^r^, Cm^r^, Amp^r^ | This study | |
| YKY053 (Δ*rpoS/*P*_1580_-luxAB*) | Δ*rpoS* containing pUTat-P*_1580_*-*luxAB*, Col^r^, Str^r^, Cm^r^, Amp^r^ | This study | |
| YKY054 (WT*/*P*_1580 mut_-luxAB*) | Wild-type containing pUTat-P*_1580 mut_*-*luxAB*, Col^r^, Str^r^, Cm^r^, Amp^r^ | This study | |
| YKY055 (WT::P*_eseB_*-*luc*) | Wild-type, P*_eseB_-luc* in the neutral position, containing pUTat, Col^r^, Str^r^, Cm^r^_,_ Amp^r^ | This study | |
| YKY056 (Δ*rpoS*::P*_eseB_*-*luc*) | Δ*rpoS*, P*_eseB_-luc* in the neutral position, containing pUTat, Col^r^, Str^r^, Cm^r^_,_ Amp^r^ | This study | |
| YKY057 (WT::P*_evpA_*-*luc*) | Wild-type, P*_evpA_-fluc* in the neutral position, containing pUTat, Col^r^, Str^r^, Cm^r^_,_ Amp^r^ | This study | |
| YKY058 (Δ*rpoS*::P*_evpA_*-*luc*) | Δ*rpoS*, P*_evpA_-luc* in the neutral position, containing pUTat, Col^r^, Str^r^, Cm^r^_,_ Amp^r^ | This study | |
| YKY059 (WT::P*_rpoS_*-*luc*) | Wild-type, P*_rpoS_-luc* in the neutral position, containing pUTat, Col^r^, Str^r^, Cm^r^_,_ Amp^r^ | This study | |
| YKY060 (Δ*rpoS*::P*_rpoS_*-*luc*) | Δ*rpoS*, P*_rpoS_-luc* in the neutral position, containing pUTat, Col^r^, Str^r^, Cm^r^_,_ Amp^r^ | This study | |
| YKY061 (*rpoS^R99A^*::P*_eseB_*-*luc*) | *rpoS^R99A^*, P*_eseB_-luc* in the neutral position, containing pUTat, Col^r^, Str^r^, Cm^r^_,_ Amp^r^ | This study | |
| YKY062 (*rpoS^R99A^*::P*_evpA_*-*luc*) | *rpoS^R99A^*, P*_evpA_-luc* in the neutral position, containing pUTat, Col^r^, Str^r^, Cm^r^_,_ Amp^r^ | This study | |
| YKY063 (*rpoS^R99A^*::P*_rpoS_*-*luc*) | *rpoS^R99A^*, P*_rpoS_-luc* in the neutral position, containing pUTat, Col^r^, Str^r^, Cm^r^_,_ Amp^r^ | This study | |
| YKY064 (*rpoS^OE^::*P*_lac_-esrB*) | *rpoS^OE^* , replacing the original *esrB* promoter with P*_lac_* from pAKgfp1, Col^r^, Str^r^, Cm^r^ | This study | |
| YKY065 (Δ*esrB*/P*_esrB mut1_-esrB*) | Δ*esrB* containing pUTat-P*_esrB mut1_-esrB*, Col^r^, Str^r^, Cm^r^_,_ Amp^r^ | This study | |
| YKY066 (Δ*esrB*/P*_esrB mut2_*-*esrB*) | Δ*esrB* containing pUTat-P*_esrB mut2_-esrB*, Col^r^, Str^r^, Cm^r^_,_ Amp^r^ | This study | |
| YKY067 (Δ*esrB*/P*_esrB mut3_*-*esrB*) | Δ*esrB* containing pUTat-P*_esrB mut3_-esrB*, Col^r^, Str^r^, Cm^r^_,_ Amp^r^ | This study | |
| ***Escherichia coli*** |  |  | |
| YKY101 (DH5α λ*pir*) | Host for π requiring plasmids | [61] | |
| YKY102 (SM10 λ*pir*) | Host for π requiring plasmids, conjugal donor | [14] | |
| YKY103 (BL21(DE3)) | Host strain for protein expression | [13] | |
| **Plasmids** |  |  | |
| pUTat | Medium copy number cloning vector, pAT153 replicon, Amp^r^ | [16] | |
| pUTat-P*_rpsU_* | pUTat derivative containing the promoter of ETAE_0456 *(rpsU)*, Amp^r^ | Lab collection | |
| pUTat-*rpoS* | pUTat derivative containing the promoter of *rpoS* and *rpoS orf*, Amp^r^ | This study | |
| pUTat-P*_rpsU_*-*rpoS* | pUTat-P*_rpsU_* derivative containing *rpoS orf*, Amp^r^ | This study | |
| pUTat-*lon* | pUTat derivative containing the promoter of *lon* and *lon orf*, Amp^r^ |  | |
| pUTat-*flag-rpoS* | pUTat-P*_rpsU_* derivative containing the fusion of *flag*-tagged and *rpoS orf*, Amp^r^ | This study | |
| pUTat-*flag* | pUTat derivative containing the *flag*-tagged, Amp^r^ | This study | |
| pUTat-P*_esrB1_-luxAB* | pUTat derivative containing the fusion of *esrB1* promoter and *luxAB*, Amp^r^ | This study | |
| pUTat-P*_esrB2_-luxAB* | pUTat derivative containing the fusion of *esrB2* promoter and *luxAB*, Amp^r^ | This study | |
| pUTat-P*_esrB3_-luxAB* | pUTat derivative containing the fusion of *esrB3* promoter and *luxAB*, Amp^r^ | This study | |
| pUTat-P*_esrB4_-luxAB* | pUTat derivative containing the fusion of *esrB4* promoter and *luxAB*, Amp^r^ | This study | |
| pUTat-P*_esrB5_-luxAB* | pUTat derivative containing the fusion of *esrB5* promoter and *luxAB*, Amp^r^ | This study | |
| pUTat-P*_esrB6_-luxAB* | pUTat derivative containing the fusion of *esrB6* promoter and *luxAB*, Amp^r^ | This study | |
| pUTat-P*_esrB7_-luxAB* | pUTat derivative containing the fusion of *esrB7* promoter and *luxAB*, Amp^r^ | This study | |
| pUTat-P*_esrB8_-luxAB* | pUTat derivative containing the fusion of *esrB8* promoter and *luxAB*, Amp^r^ | This study | |
| pUTat-P*_esrB9_-luxAB* | pUTat derivative containing the fusion of *esrB9* promoter and *luxAB*, Amp^r^ | This study | |
| pUTat-*rpoS*^L66A^ | pUTat-P*_rpsU_* derivative containing *rpoS*^L61A^, Amp^r^ | This study | |
| pUTat-*rpoS*^R99A^ | pUTat-P*_rpsU_* derivative containing *rpoS*^R99A^, Amp^r^ | This study | |
| pUTat-*rpoS*^L61AR99A^ | pUTat-P*_rpsU_* derivative containing *rpoS*^L61AR99A^, Amp^r^ | This study | |
| pUTat-P*_sdh_*-*luxAB* | pUTat derivative containing the fusion of *sdh* promoter and *luxAB*, Amp^r^ | This study | |
| pUTat-P*_sdh mut_*-*luxAB* | pUTat derivative containing the fusion of *sdh mut* promoter and *luxAB*, Amp^r^ | This study | |
| pUTat-P*_1580_*-*luxAB* | pUTat derivative containing the fusion of the promoter of ETAE_1580 and *luxAB*, Amp^r^ | This study | |
| pUTat-P*_1580 mut_*-*luxAB* | pUTat derivative containing the fusion of the mutation promoter of ETAE_1580 and *luxAB*, Amp^r^ | This study | |
| pUTat-P*_eseB_*-*luc* | pUTat derivative containing the fusion of *eseB* promoter and *luc*, Amp^r^ | Lab collection | |
| pUTat-P*_evpA_*-*luc* | pUTat derivative containing the fusion of *evpA* promoter and *luc*, Amp^r^ | Lab collection | |
| pUTat-P*_rpoS_*-*luc* | pUTat derivative containing the fusion of *rpoS* promoter and *luc*, Amp^r^ | This study | |
| pUTat-P*_esrB mut1_-esrB* | pUTat derivative containing the fusion of *esrB mut1* promoter and *esrB orf*, Amp^r^ | This study | |
| pUTat-P*_esrB mut2_-esrB* | pUTat derivative containing the fusion of *esrB mut1* promoter and *esrB orf*, Amp^r^ | This study | |
| pUTat-P*_esrB mut3_-esrB* | pUTat derivative containing the fusion of *esrB3* promoter and *esrB orf*, Amp^r^ | This study | |
| pDMK | Suicide vector, *pir* dependent, R6K, SacBR, Km^r^,Cm^r^ | [14] | |
| pDMK-Δ*rpoS* | pDMK with ETAE_2873 fragment deleted 4 to 984 nucleotides, Km^r^,Cm^r^ | This study | |
| pDMK-P*_esrB_-kan* | pDMK with P*_esrB_-kan* fragment which will be inserted into neutral site, Km^r^,Cm^r^ | This study | |
| pDMK-P*_esrB_-luxAB* | pDMK with P*_esrB_-luxAB* fragment which will be inserted into neutral site, Km^r^,Cm^r^ | This study | |
| pDMK-P_lac_-*esrB* | pDMK with *lac* promoter fragment insert into the head of *esrB orf* on genome | This study |  |
| pDMK-P*_esrB_-luxAB* | pDMK with P*_esrB_-luxAB* fragment which will be inserted into neutral site, Kan^r^,Cm^r^ | This study |  |
| pDMK-P*_esrB mut1_-luxAB* | pDMK with P*_esrB mut1_-luxAB* fragment which will be inserted into neutral site, Kan^r^,Cm^r^ | This study |  |
| pDMK-P*_esrB mut2-_luxAB* | pDMK with P*_esrB mut2_-luxAB* fragment which will be inserted into neutral site, Kan^r^,Cm^r^ | This study |  |
| pSC189 | Suicide vector, *pir* dependent,containing mariner transposon | Lab collection |  |
| pET28a | Vector for proteins expression, Kan^r^ | Novagen |  |
| pET28a-RpoS | pET28a derivative containing *rpoS* orf, Kan^r^ | This study |  |
| pET28a-RpoS^R99A^ | pET28a derivative expressing RpoS^R99A^ variant, Kan^r^ | This study |  |
| pET28a-RpoD | pET28a derivative containing *rpoD* orf, Kan^r^ | This study |  |
