# Supplementary material for: Critical role for a promoter discriminator in RpoS control of virulence in Edwardsiella piscicida
Source: PLoS Pathog. 2018 Aug 31;14(8):e1007272. doi: 10.1371/journal.ppat.1007272 (PMC6136808; doi:10.1371/journal.ppat.1007272)
Supplement: S7 Table — (DOCX) [file ppat.1007272.s013.docx]

**Table S7 Primers used in this study**

| **Name** | **Sequence (5’ - 3’)** |
| --- | --- |
| **Mutant strains construction** | |
| *rpoS*-P1 | GAGCTCAGGTTACCCGGATCTATGAGCTATAACGGCGAAGCCT |
| *rpoS*-P2 | GCATCGCTCACATAGCTGTACCCTACCCGT |
| *rpoS*-P3 | TACAGCTATGTGAGCGATGCGGTCAAAAAA |
| *rpoS*-P4 | CCCTCGAGTACGCGTCACTAGTGGGGCCCTCTGTTCGCCACCCCTACTT |
| *rpoS*-P5 | ATAACCGCAGCTACCAGTCG |
| *rpoS*-P6 | CCCGGTCGATCGCATCTTTA |
| **Complementary and over-expression strains construction** | |
| pUTat-*rpoS-*F | CTCATCCGCCAAAACAGCCATAGGCAGAATGTTGCCTGCA |
| pUTat-*rpoS-R* | TTGGTTAAAAATTAAGGAGGTCAGCGGAACAGGGCTTCGA |
| pUTat-P*_rpsU_*-*rpoS*-F | TGAGAGGCACGAGCTCGATTATGAGTCATACTACGCTGAA |
| pUTat-P*_rpsU_*-*rpoS*-R | CTGCAGGTCGACGGATCCCCTCAGCGGAACAGGGCTTCGA |
| pUTat-*lon-*F | CTCATCCGCCAAAACAGCCAATCGTTGAAGGGACGCTGCT |
| pUTat-*lon-R* | TTGGTTAAAAATTAAGGAGGCTATTTCGCCGTCACCACGT |
| pUTat-*flag-rpoS*-PF1 | GAGAGGCACGAGCTCGATTCCGGGGATCCTCTAGATTTAAGAAGGAGATATACATATGGATTACAAGGATGACG |
| pUTat-*flag-rpoS*-PF2 | TGGATTACAAGGATGACGACGATAAGGGCGGCGGCGGCAGCGGCGGCGGCGGCAGCATGAGTCATACTACGCTGAA |
| pUTat-P*_esrB_* mut*-*P1 | CTCATCCGCCAAAACAGCCAGGCATATAAAAATATTCCAT |
| pUTat-P*_esrB_* mut*-*P2 | AAATAGTCATATTTAAAGGGTACTCCGAAT |
| pUTat-P*_esrB_* mut*-*P3 | CCCTTTAAATATGACTATTTCTATTTTGCC |
| pUTat-P*_esrB_* mut*-*P4 | TTGGTTAAAAATTAAGGAGGTTAAAACTCCAGAACCCCCA |
| **Report strains construction** | |
| P*_esrB_*-F | CTCATCCGCCAAAACAGCCAGGCATATAAAAATATTCCAT |
| P*_esrB_*-R | TCCTTATTTGTTTAAAGGGTACTCCGAATC |
| P*_esrB_*-*luxAB*-F | ACCCTTTAAACAAATAAGGAAATGTTATGA |
| P*_esrB_*-*luxAB*-R | TTGGTTAAAAATTAAGGAGGTTACGAGTGGTATTTGACGAT |
| P*_rpoS_*-F | CTCATCCGCCAAAACAGCCATAGGCAGAATGTTGCCTGCA |
| P*_rpoS_*-R | CGTCTTCCATAGCTGTACCCTACCCGTGAT |
| P*_rpoS_*-*luxAB*-F | GGGTACAGCTATGGAAGACGCCAAAAACATAA |
| P*_rpoS_*-*luxAB*-R | TTGGTTAAAAATTAAGGAGGTTACACGGCGATCTTTCCGCC |
| P*_sdh_*-F | CTCATCCGCCAAAACAGCCATAACCTCTGGTGAATGCGTG |
| P*_sdh_*-R | TCCTTATTTGCTGGGGCGGTATCAAGCAGC |
| P*_sdh_*-*luxAB*-F | TCCGCCCCAGCAAATAAGGAAATGTTATGA |
| P*_sdh_*-*luxAB*-R | TTGGTTAAAAATTAAGGAGGTTACGAGTGGTATTTGACGAT |
| P_1580_-F | CTCATCCGCCAAAACAGCCACCGTACTCCGTTACTATTAT |
| P_1580_-R | TCCTTATTTGTATATTCCCAGATTTTTTGT |
| P_1580_-*luxAB*-F | TGGGAATATACAAATAAGGAAATGTTATGA |
| P_1580_-*luxAB*-R | TTGGTTAAAAATTAAGGAGGTTACGAGTGGTATTTGACGAT |
| P*_esrB_-kan*-P1 | GAGCTCAGGTTACCCGGATCTATACAGCACAGCTGACGGTATTGCTGATGCTG |
| P*_esrB_-kan*-P2 | TTTATATGCCATCGCGCTTTTATTCTACGGTAACC |
| P*_esrB_-kan*-P3 | AAAGCGCGATGGCATATAAAAATATTCCAT |
| P*_esrB_-kan*-P4 | GTTCAATCATTTTAAAGGGTACTCCGAATC |
| P*_esrB_-kan*-P5 | ACCCTTTAAAATGATTGAACAAGATGGATTGCACG |
| P*_esrB_-kan*-P6 | TAAGCATCCCTCAGAAGAACTCGTCAAGAAGGCGA |
| P*_esrB_-kan*-P7 | GTTCTTCTGAGGGATGCTTAGGCATCCCCTTTTTA |
| P*_esrB_-kan*-P8 | CCCTCGAGTACGCGTCACTAGTGGGGCCCTTATAAGATATACAGGAGAAGCCCTG |
| P*_esrB_-luxAB*-P1 | GAGCTCAGGTTACCCGGATCTATACAGCACAGCTGACGGTATTGCTGATGCTG |
| P*_esrB_-luxAB*-P2 | TTTATATGCCATCGCGCTTTTATTCTACGGTAACC |
| P*_esrB_-luxAB*-P3 | AAAGCGCGATGGCATATAAAAATATTCCAT |
| P*_esrB_*-*luxAB*-P4 | TCCTTATTTGTTTAAAGGGTACTCCGAATC |
| P*_esrB_*-*luxAB*-P5 | ACCCTTTAAACAAATAAGGAAATGTTATGA |
| P*_esrB_*-*luxAB*-P6 | TAAGCATCCCTTACGAGTGGTATTTGACGAT |
| P*_esrB_*-*luxAB*-P7 | CCACTCGTAAGGGATGCTTAGGCATCCCCTTTTTA |
| P*_esrB_-luxAB*-P8 | CCCTCGAGTACGCGTCACTAGTGGGGCCCTTATAAGATATACAGGAGAAGCCCTG |
| P*_lac_*-*esrB*-P1 | GAGCTCAGGTTACCCGGATCTATCATGTCCTTCACCCGGAACA |
| P*_lac_*-*esrB* -P2 | TTAAATCTAGTGTCATGCGATTGGGAGGTC |
| P*_lac_*-*esrB* -P3 | TCGCATGACACTAGATTTAAGAAGGAGATATACAT |
| P*_lac_*-*esrB* -P4 | AAATAGTCATTCTCATCCGCCAAAACAGCC |
| P*_lac_*-*esrB* -P5 | GCGGATGAGAATGACTATTTCTATTTTGCC |
| P*_lac_*-*esrB* -P6 | CCCTCGAGTACGCGTCACTAGTGGGGCCCTTTAAAACTCCAGAACCCCCA |
| Luc-insert-P1 | GAGCTCAGGTTACCCGGATCTATACAGCACAGCTGACGGTATTGCTGATGCTG |
| Luc-insert-P2 | GGCGGATGAGATCGCGCTTTTATTCTACGGTAACC |
| Luc-insert-P3 | AAAGCGCGATCTCATCCGCCAAAACAGCCA |
| Luc-insert-P4 | TAAGCATCCCTTGGTTAAAAATTAAGGAGGTTAC |
| Luc-insert-P5 | TTTTAACCAAGGGATGCTTAGGCATCCCCTTTTTA |
| Luc-insert-P6 | CCCTCGAGTACGCGTCACTAGTGGGGCCCTTATAAGATATACAGGAGAAGCCCTG |
| ***In vitro* transcription** | |
| *esrB-frag-F* | GCGAATAAAACAACCATTCA |
| *esrB-frag-R* | CGGTAGCGGGGCTGGCTGTC |
| *esrB-frag-NF* | AGTGTGCCACTGATTCGGAG |
| **Protein expression** | |
| *rpoS*-EF | CAGCAAATGGGTCGCGGATCCATGAGTCATACTACGCTGAA |
| *rpoS*-ER | GTGGTGCTCGAGTGCGGCCGCTCAGCGGAACAGGGCTTCGA |
| *rpoD*-EF | CAGCAAATGGGTCGCGGATCCATGGAGCAAAACCCGCAGTC |
| *rpoD*-ER | GTGGTGCTCGAGTGCGGCCGCTTAGTCGTCCAGGAAGCTGC |
| **EMSA** | |
| P*_esrB_*-SF | TTACCCTCAGCAAAATGCTA |
| P*_esrB_*-SR | TATTTAAAGGGTACTCCGAA |
| P*_rpoS_*-SF | GGGCGGGTGGTCTATGCCGG |
| P*_rpoS_*-SR | AGCTGTACCCTACCCGTGAT |
| P*_mdtJ_*-SF | ATGAAAACGCATAATCGTTT |
| P*_mdtJ_*-SR | ATTATTTCTCCACTTATTTA |
| P*_sdhC_*-SF | TAACCTCTGGTGAATGCGTG |
| P*_sdhC_*-SR | CTGGGGCGGTATCAAGCAGC |
| P*_bglG_*-SF | TCGGCGCGGTGATCGCCAAC |
| P*_bglG_*-SR | CCCCCGGGCGCGCTCCCGCC |
| P*_hslJ_*-SF | ATACCATCGCCATTCGCACA |
| P*_hslJ_*-SR | GTCAGCTTCTCTCTCCAGAG |
| P*_uspB_*-SF | GAATCCCCATACCCATGGTG |
| P*_uspB_*-SR | GGTTTCCCCTCCCGGCATCC |
| *gyrB*-SF | CCGATGATGGTACGGGTCTG |
| *gyrB*-SR | GCTTTTCAGACAGGGCGTTC |
| P_0786_-SF | CAATCGGCTTTTTGTCGTGT |
| P_0786_-SR | TTTTAATGTCCTTGATATGG |
| P_1218_-SF | CTATTCGGTGGGGTGTGGGG |
| P_1218_-SR | AGAGGACTCCTGGCGTCTGG |
| P_2647_-SF | CGGCGTCCCCGGCAAAGTCG |
| P_2647_-SF | GATGGGGACTACAACATCCG |
| P_1580_-SF | CCGTACTCCGTTACTATTAT |
| P_1580_-SF | TATATTCCCAGATTTTTTGT |
| **ChIP** | |
| P*_esrB_*-EF | CGAAATTTGAATACCGACGA |
| P*_esrB_*-ER | TATTTAAAGGGTACTCCGAA |
| **qRT-PCR** | |
| RT-*esrB*-F | GGGCTTTGCTTCAGGACGTA |
| RT-*esrB*-R | GCCCGATCTGGTCTTACTCG |
| RT-*eseB*-F | CCCCTTTATCCAGCCCCTTG |
| RT-*eseB*-R | GCCAAGTTCAAGAAAGCGGG |
| RT-*eseC*-F | CGATGGCGGAGAAAATCCCT |
| RT-*eseC*-R | ACAACGCCAATCTCAAAGCG |
| RT-*eseD*-F | GCCGTTGCTAATGGTATGCG |
| RT-*eseD*-R | TGCGCCAGTATCAACAGACA |
| RT-*evpP*-F | GAATGGGGACGACTCACCTC |
| RT-*evpP*-R | AAATCCACCGAACCAGGCAT |
| RT-*evpA*-F | ATCTGTCATTCCGCACCGAG |
| RT-*evpA*-R | TTTTCAGGTCAGAGAGGCGG |
| RT-*evpC*-F | GGTAAGGCGATGATGTCGGT |
| RT-*evpC*-R | GTGGCCCCTGAGCATTGATA |
| RT-*gyrB*-F | CCGATGATGGTACGGGTCTG |
| RT-*gyrB*-R | GCTTTTCAGACAGGGCGTTC |
| RT-*lon*-F | CGATCTGTCCGACGTGATGT |
| RT-*lon*-R | GGCGCTATCATCCACCGTTA |
| RT-*rpoS*-F | GCGGTACGCAGATAGACGTT |
| RT-*rpoS*-R | CTTTTCGACCTACGCCACCT |
| RT-P*_esrB_*-F | TCGCTCGGGGCATTTGAATA |
| RT-P*_esrB_*-R | CAGCAGTTTTGCTTGACCGT |
| RT-1584-F | TTTCTGACGGATAGTGGCCG |
| RT-1584-R | GGTTCCCATCGACCTCACTG |
| RT-1770-F | CATCTGCCCTTTTCCCCAGT |
| RT-1770-R | GGGTAAAACCGATGCCCAGA |
| RT-*gtrA*-F | TGAATACCGCCTTGCACTGG |
| RT-*gtrA*-R | AAAACGAAAAGGTGACGGCG |
| RT-1947-F | GTCGATACGCAGGGAGAGTG |
| RT-1947-R | TGCGACAAACAGGTCGTACA |
| RT-*bglG2*-F | GGATTCACTCATGCCCTGCT |
| RT-*bglG2*-R | GGCTGTGGCTGTGATTCTTG |
| RT-*uspB*-F | GTGGCTGGTAAAGAAACCGC |
| RT-*uspB*-R | TCGCGTTATTTTGGGCGTTG |
| RT-1580-F | GAGGGGATTGGCATCCTGTT |
| RT-1580-R | TGCGTCCCTTTTTCACCGTA |
| RT-*sdhC*-F | GTCAGGATACCCCACAGCAC |
| RT-*sdhC*-R | GATTGCGTCGATCTTGCACC |
| RT-*csrA*-F | TTTCGGAGCATTCACACCGA |
| RT-*csrA*-R | TGTTGGGGGTGAAGGGAAAC |
| **Sequencing** | |
| **Adaptor biosynthesis** | |
| AD_fork truncated NH2 | TACCACGACCA |
| AD_Index Fork R | GTGACTGGAGTTCAGACGTGTGCTCTTCCGATCTGGTCGTGGTAT |
| **1^st^ PCR** | |
| Seq-out (pSC189) | ACAATTCGTTCAAGCCGAGAT |
| Index ‘R’ primer | GTGACTGGAGTTCAGACGTGTG |
| **2^nd^ PCR** | |
| P5-InvRep-Var1F | AATGATACGGCGACCACCGAGATCTACACTCTTTCCCTACACGACGCTCTTCCGATCTGACTTATCAGCCAACCTGT |
| P5-InvRep-Var2F | AATGATACGGCGACCACCGAGATCTACACTCTTTCCCTACACGACGCTCTTCCGATCTCGACTTATCAGCCAACCTGT |
| P5-InvRep-Var3F | AATGATACGGCGACCACCGAGATCTACACTCTTTCCCTACACGACGCTCTTCCGATCTATGACTTATCAGCCAACCTGT |
| P5-InvRep-Var4F | AATGATACGGCGACCACCGAGATCTACACTCTTTCCCTACACGACGCTCTTCCGATCTTGTCGACTTATCAGCCAACCTGT |
| P5-InvRep-Var5F | AATGATACGGCGACCACCGAGATCTACACTCTTTCCCTACACGACGCTCTTCCGATCTTCGACGACTTATCAGCCAACCTGT |
| P5-InvRep-Var6F | AATGATACGGCGACCACCGAGATCTACACTCTTTCCCTACACGACGCTCTTCCGATCTGCAGCGACGACTTATCAGCCAACCTGT |
| P7-AD001-index-R | CAAGCAGAAGACGGCATACGAGATCGTGATGTGACTGGAGTTCAGACGTGTGCTCTTCCGATC |
| **qRT-PCR** | |
| P5-F | AATGATACGGCGACCACCGAGATCT |
| P7-R | CAAGCAGAAGACGGCATACGAGAT |
